# Factors Influencing the Antifolate Activity of Synthetic Tea-Derived Catechins

**DOI:** 10.3390/molecules18078319

**Published:** 2013-07-16

**Authors:** Magalí Sáez-Ayala, María Piedad Fernández-Pérez, Soledad Chazarra, Nani Mchedlishvili, Alberto Tárraga-Tomás, José Neptuno Rodríguez-López

**Affiliations:** 1 Department of Biochemistry and Molecular Biology A, School of Biology, Regional Campus of International Excellence “Campus Mare Nostrum”, University of Murcia, 30100, Murcia, Spain; E-Mails: magali@um.es (M.S.-A.); mapy3212@hotmail.com (M.P.F.-P.); solecha@um.es (S.C.); 2 Durmishidze Institute of Biochemistry and Biotechnology of Agrarian University of Georgia, 0131, Tbilisi, Georgia; E-Mail: nanamchedl@hotmail.com; 3 Department of Organic Chemistry, Faculty of Chemistry, Regional Campus of International Excellence “Campus Mare Nostrum”, University of Murcia, 30100, Murcia, Spain; E-Mail: atarraga@um.es

**Keywords:** tea catechins, dihydrofolate reductase, antifolates, albumin, drug design

## Abstract

Novel tea catechin derivatives have been synthesized, and a structure-activity study, related to the capacity of these and other polyphenols to bind dihydrofolate reductase (DHFR), has been performed. The data showed an effective binding between all molecules and the free enzyme, and the dissociation constants of the synthetic compounds and of the natural analogues were on the same order. Polyphenols with a catechin configuration were better DHFR inhibitors than those with an epicatechin configuration. Antiproliferative activity was also studied in cultured tumour cells, and the data showed that the activity of the novel derivatives was higher in catechin isomers. Derivatives with a hydroxyl group *para* on the ester-bonded gallate moiety presented a high *in vitro* binding to DHFR, but exhibited transport problems in cell culture due to ionization at physiologic pHs. The impact of the binding of catechins to serum albumin on their biological activity was also evaluated. The information provided in this study could be important for the design of novel medicinal active compounds derived from tea catechins. The data suggest that changes in their structure to avoid serum albumin interactions and to facilitate plasmatic membrane transport are essential for the intracellular functions of catechins.

## 1. Introduction

**Figure 1:**
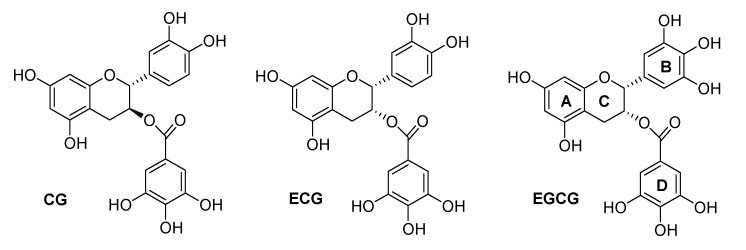
Flavonoids present in tea with epicatechin (ECG, EGCG) and catechin configurations (CG).

Tea is the most popular beverage, consumed by two-thirds of the World’s population. Among all teas, green tea is the most studied for its reputed health benefits [1]. Tea polyphenols are considered to contribute to preventive effects against various pathological disorders, including cancer. The major components of tea are catechins, which contain a benzopyran skeleton with a phenyl group substituted at the 2-position and a hydroxyl (or ester) function at the 3-position. Variations in the catechin structure include the stereochemistry of the 2,3-substituents and the number of hydroxyl groups in the B-ring and D-ring (Figure 1). Belonging to the flavanol class of flavonoids, the most abundant catechins found in tea leaves include epigallocatechin-3-gallate [EGCG], epigallocatechin [EGC], epicatechin-3-gallate [ECG], epicatechin [EC], catechin-3-gallate [CG], and gallocatechin-3-gallate [GCG] (Figure 1). Several *in vivo* experiments have shown the chemo-preventative effects of EGCG against all stages of carcinogenesis in animal models of breast, lung, skin, prostate, and colon cancers [2,3]. There has been extensive investigation into the mechanism by which EGCG might act in cancer prevention, and recent attempts to define the molecular action mechanism of green tea components have found that EGCG affects several molecular targets and carcinogenesis pathways. The 3-gallyl moiety of catechins is essential for the inhibition of several of their proposed enzymatic targets, such as the proteasome [4], 5-cytosine DNA methyltransferase 1 (DNMT1) [5], glutamate dehydrogenase [6], and dihydrofolate reductase (DHFR) [7]. Recently, we reported that the ester-bound gallate catechins isolated from green tea are potent inhibitors of DHFR activity at concentrations found in the serum and tissues of green tea drinkers [7]. Since this first report describing the antifolate activity of tea polyphenols, several studies by us and by other research groups have confirmed this activity [8,9,10] and have reported that EGCG inhibits DHFR from a variety of biological sources [11,12,13,14]. 

However, the excellent anticancer properties of tea catechins are significantly limited by their poor bioavailability, which is related to their low stability in neutral or slightly alkaline solutions, and by their inability to easily cross cellular membranes [15]. In an attempt to solve these bioavailability problems, we synthesized a 3,4,5-trimethoxybenzoyl analogue of ECG (TMECG) that exhibits high antiproliferative activity against several cancer cell lines, especially melanoma [16]. Methoxy protection of the reactive hydroxyl groups in the D-ring of ECG yielded a derivative stable under cell culture conditions that is more efficiently transported through the cell membranes than its natural tea parent catechin [16]. Because the major polyphenols present in tea have epicatechin configurations, many of the studies designed to elucidate the biological activities of these tea catechins have been performed with epicatechin derivatives, but catechin gallate (CG) also inhibits the proliferation of cancer cells derived from human oral cavity tissues [17]. As part of our on-going efforts to develop new tea-derived compounds, we synthesized a trimethoxybenzoyl analogue of CG (TMCG) [18]. This compound shared bioavailability advantages with its epimer TMECG, inhibited growth, and induced apoptosis in several human cancer cell lines. TMCG exhibited characteristics of a slow-binding inhibitor of DHFR; an explanation for the irreversibility of the slowly dissociating enzyme complex was provided by molecular modelling [18].

In an attempt to explore the structure-activity relationship of natural and synthetic polyphenols as inhibitors of DHFR, several new derivatives with different stereochemistry and D-ring substitutions were designed and synthesized. This allowed us to compare the epimeric differences between epicatechin and catechin configurations and the different substitutions of hydroxyl groups on the D-ring with respect to DHFR binding and antiproliferative actions against cancer cells. We achieved the synthesis of derivatives with methoxy groups in the *meta* position on the esterified D-ring in both catechin and epicatechin configurations. We also synthesized the fully acetylated derivative of this catechin isomer and its monoacetylated derivative with a hydroxyl group *para* on the D-ring. The factors affecting the biological activities of these synthetic polyphenols, including their interaction with serum albumin, were studied.

## 2. Results and Discussion

### 2.1. Synthesis

**Scheme 1:**
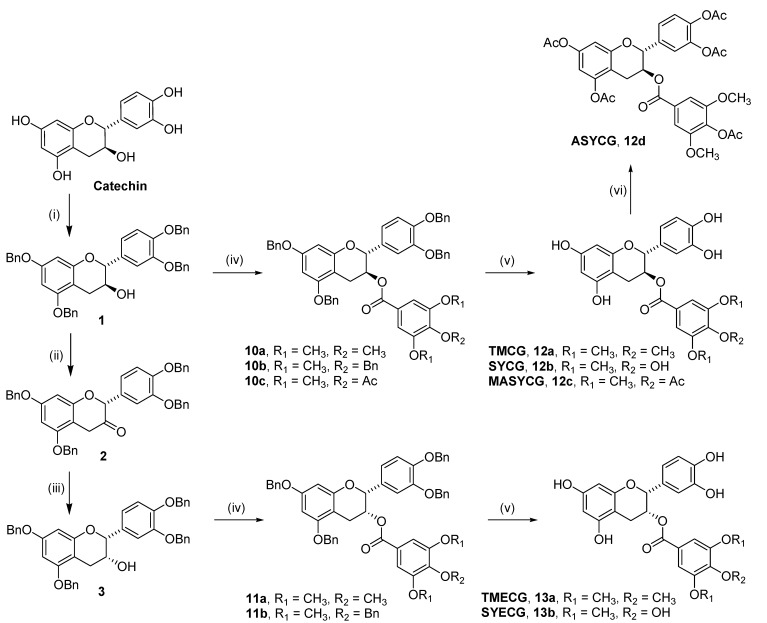
Synthesis of the tea catechin derivatives **12a**–**d** and **13a**–**b**.

The syntheses of TMECG (**13a**) and TMCG (**12a**) was carried out starting from the commercially available catechin, following a procedure previously described by our research group [16,18]. Consequently, this general route was also used to prepare the new compounds **12b**–**d**, **13b**. The reaction sequence was designed to avoid problems associated with unspecific blockage of the 3-hydroxy group of epicatechin [19,20]. Therefore, all compounds (both catechin and epicatechin configurations) were synthesized starting from catechin following the multi-step reaction sequence shown in Scheme 1, which involved an inversion of configuration at C-3 through an oxidation–reduction process to obtain compounds with an epicatechin configuration. After selectively protecting the phenolic hydroxyl groups of catechin and epicatechin, the resulting compounds were treated with the appropriate acid halides to give rise to the corresponding esters which, in a final step, undergo deprotection of the phenolic groups by treatment with H_2_ in the presence of 10% Pd/C. 

**Scheme 2:**
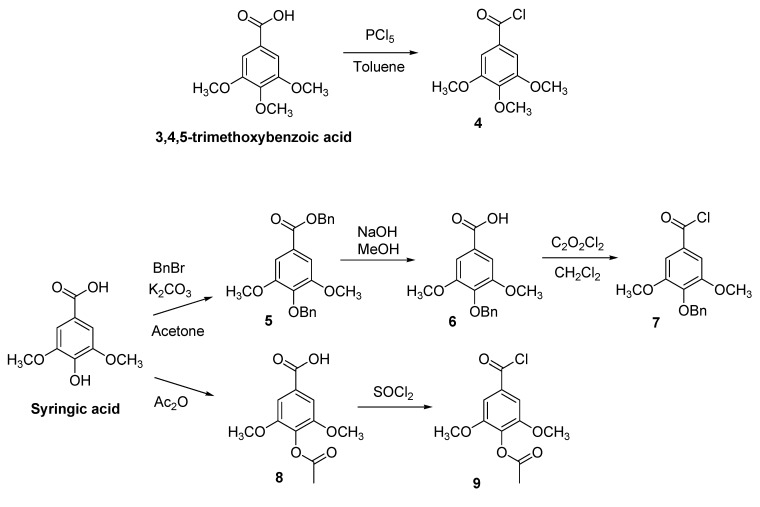
Synthesis of acid chlorides **4**, **7** and **9**.

It is worth noting that for introducing two methoxy groups *meta* to the ester-bonded gallate moiety, such as in SYCG (**12b**) and SYECG (**13b**), we used the 4-(benzyloxy)-3,5-dimethoxybenzoyl chloride (**7**), which was prepared from the commercially available syringic acid (3,5-dimethoxy-4-hydroxybenzoic acid) in a three step sequence: (i) treatment with benzyl bromide in the presence of K_2_CO_3_ to give **5**; (ii) hydrolysis of the benzyl ester group to the corresponding carboxylic acid derivative **6**; and iii) treatment of **6** with oxalyl chloride to yield **7** (Scheme 2). Thus, reaction of **7** with the isomers **1** and **3** in the presence of DMAP (dimethylaminopyridine), yielded the corresponding esters **10b** and **11b **which by hydrogenolysis underwent cleavage of the benzyl ether functionalities to produce the final compounds **12b** and **13b** in high yield and purity. 

*Reagents and conditions*: (i) BnBr, K_2_CO_3_, DMF, −10 °C to RT, overnight; (ii) Dess–Martin periodinane, CH_2_Cl_2_, RT, 3 h; (iii) l-Selectride, *n*Bu_4_NCl, THF, −78 °C, 3 h; (iv) **4**, **7** or **9**, CH_2_Cl_2_, DMAP, RT, 18 h; (v) H_2_, 10% Pd/C, THF/MeOH (3:1), RT, 15 h.

To synthesize the fully acetylated derivative ASYCG (**12d**), compound **12b** was peracetylated by reaction with acetic anhydride in the presence of pyridine. However, the synthesis of MASYCG (**12c**) bearing only one acetyl group in the *para* position to the ester-bonded gallate moiety, required the previous acetylation of the syringic acid to give **8**, which on treatment with thionyl chloride in CH_2_Cl_2_ gave rise to 4-acetoxy-3,5-dimethoxybenzoyl chloride (**9**, Scheme 2). Thus, treatment of **1** with the acid chloride **9** in CH_2_Cl_2_ and in the presence of DMAP, yielded **10c**, which undergoes hydrogenolysis to give **12c** in high yield and purity (Scheme 1). 

All compounds were characterized by mono- and bi-dimensional ^1^H- and ^13^C-NMR and mass spectrometry techniques (see Supplementary Materials).

### 2.2. Biological Activities

#### 2.2.1. Binding of polyphenols to DHFR

Natural and synthetic derivatives of tea catechins have shown to be good inhibitors of human DHFR [7,11,16,18]. To determine the ability of natural and synthetic polyphenols to bind DHFR, we performed a structure-activity study by analyzing the binding of these polyphenols to DHFR using fluorescence quenching. Effective binding to free recombinant human DHFR (rHDHFR) was determined by measuring the decrease in enzyme fluorescence that occurs upon the formation of the enzyme-inhibitor complex. When DHFR fluorescence was excited at 290 nm, its emission spectra showed a maximum at 340–350 nm. The binding of inhibitors quenched this fluorescence, and the data showed an effective union between all molecules and the free enzyme (Table 1). The dissociation constants of the synthetic compounds were on the same order as those of the natural analogues, indicating the high DHFR binding capacities of these new derivatives. 

**Table 1:** Dissociation constants of free rHDHFR for natural and synthetic catechins. * *p* < 0.05 with respect to their respective catechin isomers.

Compound	K_D_ (μM)
CG	0.56 ± 0.07
ECG	0.81 ± 0.09 *
TMCG	0.9 ± 0.1
TMECG	2.1 ± 0.2 *
SYCG	0.72 ± 0.10
SYECG	0.77 ± 0.07

In general, the catechin isomers bound to the enzyme with a lower dissociation constant than the epicatechin isomers, indicating that the polyphenols with a catechin configuration (CG, TMCG, and SYCG) had better binding capacity than those with an epicatechin configuration (ECG, TMECG, and SYECG). This result also indicates that the *cis* disposition of the lateral esterified ring interferes with binding compared to the *trans* disposition. Moreover, we studied the influence of different positions of methoxy groups on the ester-bonded gallate moiety on the binding to DHFR. We analyzed natural compounds (three hydroxyl groups), trimethoxy-derivatives (three methoxy groups in the *meta* and *para* positions), and new derivatives of syringic acid (two methoxy groups *meta* and one hydroxyl group *para*) (Table 2). These results showed that the presence of methoxy groups in ring D hindered the binding of polyphenols to DHFR and that one hydroxyl group in the *para* position on lateral ring D was important for the stabilization of the enzyme-inhibitor complex. 

**Table 2:** Order of the binding strengths of catechins to rHDHFR depending on the substitution in the D-ring. * *p* < 0.05 with respect to the binding of natural compounds to DHFR. 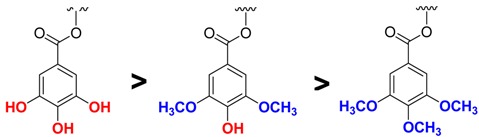

(K_D_, μM)			
Catechin	0.56	0.72	0.90 *
Epicatechin	0.81	0.77	2.10 *

#### 2.2.2. Cancer Cell Antiproliferative Activity

Antifolate compounds are designed to inhibit DHFR and act specifically during DNA and RNA synthesis, making them more toxic to rapidly dividing cells. Recent evidences indicate that tea-catechins and derived compounds could inhibit DHFR and block the folate cycle in cancer cells [16,18]. Although inhibition of DHFR by these compounds could be a plausible explanation for their antifolate activity, the possibility of these compounds targeting (folate)-enzymes other than DHFR could not be excluded. To determine whether these newly synthetized compounds disrupt the folate cycle in cell culture, we studied their antiproliferative activity in the melanoma cell line SK-MEL-28 (Table 3). Concentration- and time-dependent effects were analyzed using the colorimetric 4,5-(3)-dimethylthiazol-2-yl)-2,5-diphenyltetrazolium bromide (MTT) method [18]. 

**Table 3:** Antiproliferative effects of natural and synthetic catechins in melanoma cells. Half-maximal inhibitory concentration (IC_50_) of compounds against the SK-MEL-28 melanoma cell line after 6 days of treatment. * *p* < 0.05 when compared with their respective fully methylated polyphenols in ring D (TMCG and TMECG, respectively). ** *p* < 0.05 when compared with SYCG.

Compound	IC_50_ (μM)
CG	82.2 ± 8.0
ECG	> 100
TMCG	1.5 ± 0.4
TMECG	2.5 ± 0.6
SYCG	126.3 ± 10.0 *
SYECG	198.9 ± 12.0 *
MASYCG	60.9 ± 6.0 **
ASYCG	37.4 ± 4.0 **

The results showed that all compounds caused a decrease in the number of viable cells depending on the concentration and time. Previous results indicated that the reduced viability of cancer cells in the presence of TMECG or TMCG was indeed due to apoptosis induction [16,18]. Compounds with a catechin configuration presented higher antiproliferative activity than those with an epicatechin configuration (*p* < 0.05), as observed in *in vitro* enzymatic assays with DHFR. However, the two compounds with one hydroxyl group *para* on a lateral esterified ring D (SYCG and SYECG), that bind tightly to DHFR *in vitro*, did not show the expected antiproliferative activity. To verify that this effect was not due to a particular cell line, we carried out the same assays in other cell lines of melanoma, colon, and breast cancer with SYCG (data not shown), which proved that this effect was a general effect. Although SYCG and SYECG were stronger inhibitors of DHFR than the other compounds (as observed by binding studies), they showed a reduced efficacy in inhibiting the growth of tumour cells. This effect might be a result of a problem in cellular transport. Although they could be good inhibitors of DHFR, these compounds cannot cross the plasma membrane to reach their cellular target. 

#### 2.2.3. pH-Related Effects on Catechin Membrane Transport

**Figure 2:**
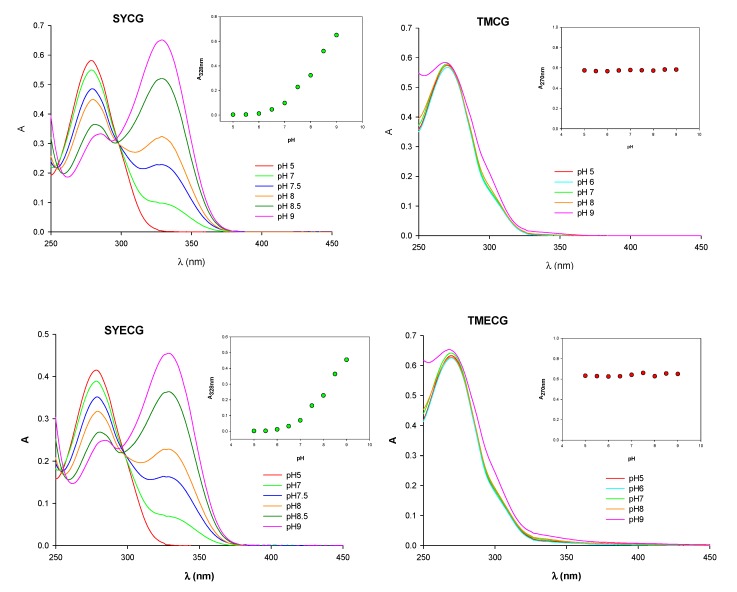
Effect of pH on the ionization state of SYCG, SYECG, TMCG, and TMECG. UV-visible absorption spectra of the compounds at different pH values. The inset is a representation of A_328/270_ *vs.* pH.

Importantly, recent studies have presented data suggesting that pH could be of relevance to tea catechin transport and to their biological activity [11,21]. These results led us to study the effect of pH on the protonation state of the inhibitors of DHFR. It has been shown that the protonation state of methotrexate, trimethoprim, and pyrimethamine modulates the binding of these antifolates to DHFRs from several sources [22]. The absorption spectra of SYCG and SYECG are strongly affected by the pH (Figure 2). The absorbance maximum at 278 nm undergoes a bathochromic displacement to 328 nm, which is greatest at alkaline pH (pH > 9.0). However, the absorption spectra of the trimethoxylated derivatives, TMCG and TMECG, showed no significant changes in the pH range of 5.0–9.0 (Figure 2). 

**Scheme 3:**
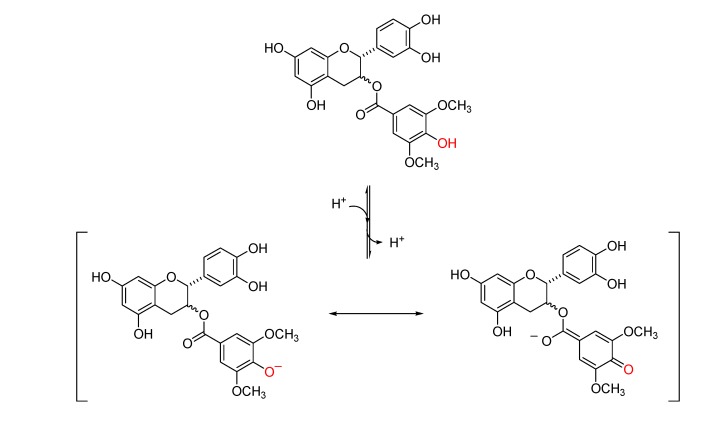
Schematic representation of the effect of pH on SYCG and SYECG ionization showing the proposed protonated and deprotonated species.

To determine if the spectral changes in the SYCG and SYECG solutions with pH were due to an irreversible oxidation reaction or to a reversible protonation step, a pH titration experiment was carried out. An alkaline solution of SYCG and SYECG (pH 8.5) was acidified to pH 2.5 by the addition of dilute HCl. A change in the spectra was observed, indicating a reversible protonation reaction. The spectral changes with pH observed in SYCG and SYECG can be attributed to the hydroxyl group *para* on the ester-bonded gallate moiety. At acidic pH, these derivatives are mainly present as protonated species, whereas at basic pH or values near neutrality, these derivatives evolve toward the deprotonated species, which are stabilized by resonance (Scheme 3). At physiologic pH, both SYCG and SYECG are partially in their ionic form, which could be responsible for their limited traffic through the plasma membrane and thereby account for their low antiproliferative activities. As described, an important factor that decreases catechin bioavailability might be their inability to easily cross cellular membranes by passive diffusion. The presence of several hydroxyl groups in these molecules gives them a high hydrophilic character, which increases the difficulty of passing across lipid membranes.

For the TMCG and TMECG derivatives, the presence of three methoxy groups increases the hydrophobic character, so they are easily transported [16], which favours their anti-tumour activities with respect to natural catechins, despite being weaker inhibitors of DHFR. However, SYCG and SYECG present an additional inconvenient related with the overall negative charges at physiological pH, which hinder their ability to cross the cellular membrane. To test this hypothesis and to prevent the ionization of this hydroxyl group to facilitate their transport into the cell, we synthesized two derivatives from the structure of SYCG with fully or partially acetylated hydroxyl groups. 

**Figure 3:**
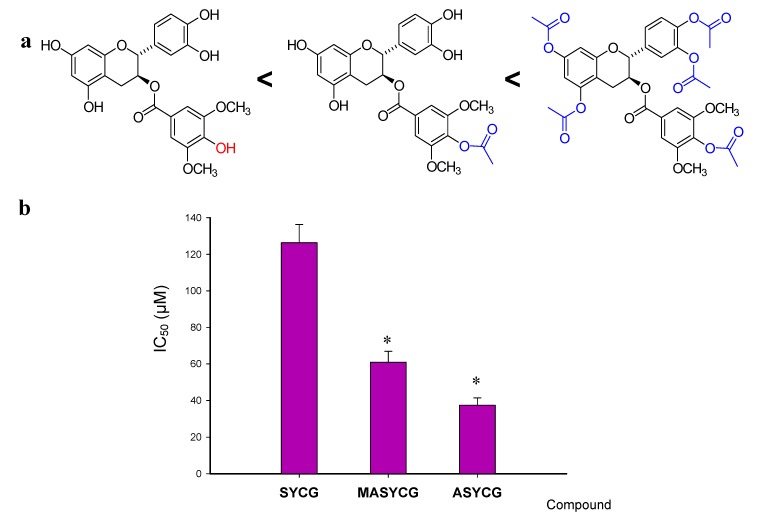
(**a**) Influence of the acetylation of hydroxyl groups of SYCG on the antiproliferative activity in melanoma cells. (**b**) Half-maximal inhibitory concentration (IC_50_) of compounds against the SK-MEL-28 melanoma cell line after six days of treatment. * *p* < 0.05 when compared with SYCG.

Esters are the most common pro-drugs used, and it is estimated that 49% of all marketed pro-drugs are activated by enzymatic hydrolysis [23]. Ester pro-drugs are produced by masking the polar groups, resulting in an enhanced lipophilicity and in the protection of the hydroxyl groups from ionization, biotransformation or oxidative degradation. Therefore, we synthesized two derivatives of SYCG as potential pro-drugs. After enzymatic hydrolysis of the ester bound inside cells, SYCG will be recovered to perform its antifolate activity. We synthesized the fully acetylated derivative (ASYCG) and the monoacetylated derivative of the hydroxyl group *para* on the ester-bonded gallate moiety (MAYCG) and studied their antiproliferative activity in SK-MEL-28 by analyzing concentration- and time-dependent effects (Figure 3). The presented data indicate that the anti-tumour activities of these pro-drugs increased with respect to that of SYCG. The fully acetylated derivative was more active than the partially acetylated derivative (ASYCG > MASYCG). This effect could be partially explained by the fact that ASYCG lacks hydroxyl groups, facilitating its transport into the cell. Although the antiproliferative activity of the monoacetylated derivative improved with respect to that of SYCG, the numerous hydroxyl groups in the molecule may still complicate its transport across the plasma membrane. All together, the results showed improved transport of these acetylated compounds into the cells, but these effects were lower than expected because they did not reflect the strong *in vitro* binding to DHFR by SYCG, which was higher than that of TMCG. This effect could be a result of an incomplete enzymatic hydrolysis of acetyl groups, which hinder DHFR from binding to the free hydroxyl groups of the molecule because of a low recovery of OH groups. 

#### 2.2.4. Influence of Catechin-Serum Albumin Interactions on Polyphenol Activity

It is widely accepted that pharmacokinetic analyses in plasma do not accurately determine the overall levels of catechins because polyphenols are known to partition into blood cells and plasma proteins. This bound pool may be considered as a “reservoir”, partially protected from conjugative enzymes and thus with a potentially extended lifetime, which can replace the molecules eliminated via metabolism and excretion. Specifically, recent studies have suggested that tea catechins form complexes with human serum albumin (HSA) for transport in human blood, and their binding affinity for albumin is believed to modulate their bioavailability [24]. Recently, it has been demonstrated that the affinity of tea catechins for albumin varies according to their chemical structure [24,25]. Thus, galloylated catechins have higher binding affinities with HSA than non-galloylated catechins, and the binding affinities of several methylated derivatives were lower than that of EGCG [24]. In agreement with these previous data, we have observed that TMECG has an intermediate binding affinity to HSA (K_A_ = 4.2 × 10^4^ M^−1^) compared with that of EGCG (K_A_ = 2.6 × 10^5^ M^−1^) or EC (K_A_ = 3.2 × 10^3^ M^−1^). However, it must be kept in mind that, although the intrinsic affinity of TMECG for albumin may be weaker than that of EGCG, the physiologic concentration of serum albumin (~0.6 mmol/L) is most likely large enough to allow the extensive binding of TMECG to this serum protein. 

Although the conventional view is that cellular uptake is proportional to the unbound concentration of metabolites, the effect of albumin binding on the biological activity of polyphenols is unclear. On the one hand, the incubation of quercetin in human whole blood or in suspensions of erythrocytes in the absence of plasma proteins suggests that binding to albumin could considerably decrease the association of quercetin with these cells [26]. On the other hand, the cancerous cells and tissues in humans might constitute a different scenario. In this respect, albumin is believed to play an increasing role as a drug carrier in the clinical setting. Due to pathophysiological conditions in neoplastic tissue, high amounts of albumin accumulate in tumours and are metabolised by malignant cells. Primary investigations have shown that tumours metabolize substantial amounts of albumin, using it as source for nitrogen and energy [27]. Several studies with tumour-bearing animals have provided further evidence that albumin accumulates in tumours because of their altered physiology and metabolism [28]. Due to the leakiness of tumour vessels, the rate of albumin extravasation is markedly increased. Moreover, the lack of a functional lymphatic system results in the accumulation of albumin in tumour tissue. The uptake of albumin in tumour cells occurs by fluid phase endocytosis followed by lysosomal breakdown. As a result, albumin-coupled drugs are liberated within the tumour cells [28]. 

**Figure 4:**
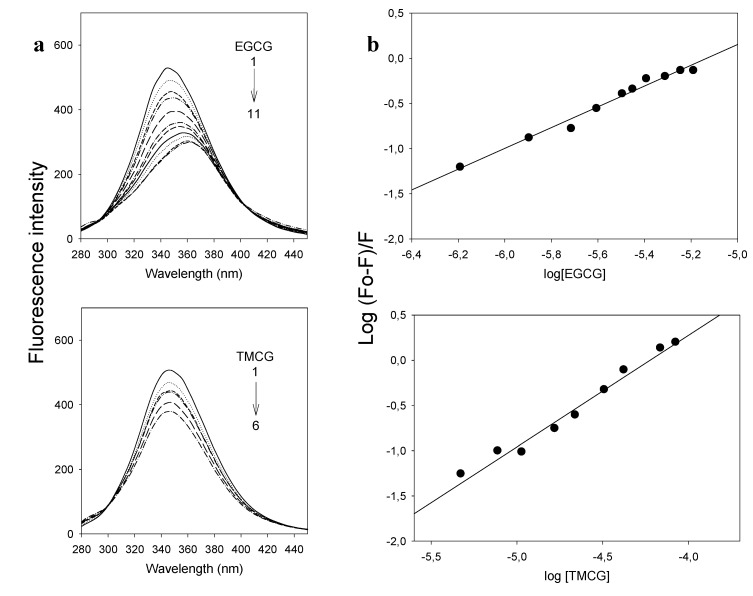
(**a**) The fluorescence emission spectra of the catechin-BSA system (T = 25 °C; λ_ex_ = 280 nm). For EGCG curves, (1–11) denote 0 to 6.5 µM, and for TMCG curves, (1–6) denote 0 to 22 µM. (**b**) Double logarithmic plots of the catechin-BSA system calculated from BSA fluorescence quenching as represented in panel **a**.

To investigate the influence of catechin-serum albumin interactions on polyphenols activity and to determine whether the degree of catechin binding to albumin may have consequences for the rate of clearance of these drugs and/or for their delivery to cancerous cells and tissues, we analyzed and compared the binding of EGCG and TMCG to bovine serum albumin (BSA). As shown in Figure 4, the fluorescence emission spectrum of BSA has an emission maximum at 350 nm when excited at 280 nm. Upon progressive additions of various concentrations of EGCG and TMCG to BSA, the emission intensity decreases, as shown in Figure 4(a). The quenching of the fluorescence intensity of BSA implies that the catechins bind at close proximity to the tryptophan and tyrosine residues and that the molecular arrangement of BSA is affected by the interactions. A progressive red shift of the fluorescence emission of BSA was observed with increasing concentration of EGCG, while no such peak shift was observed for TMCG, suggesting weaker interactions for this methylated catechin. The binding constants (K_A_) for the BSA-EGCG and BSA-TMCG complexes are 1.54 × 10^7^ M^−1^ and 1.9 × 10^5^ M^−1^, respectively (Table 4), and their plots are shown in Figure 4(b). The values of n are approximately 1 for both catechin-BSA complexes, indicating that there is only one binding site involved in each of the cases [Figure 4(b)]. In light of these findings, it can be reasoned that the binding affinity to BSA is higher for the galloylated catechin than for the methylated one, which emphasises that the three phenolic groups can enhance hydrogen bonding between BSA and EGCG [25].

**Figure 5:**
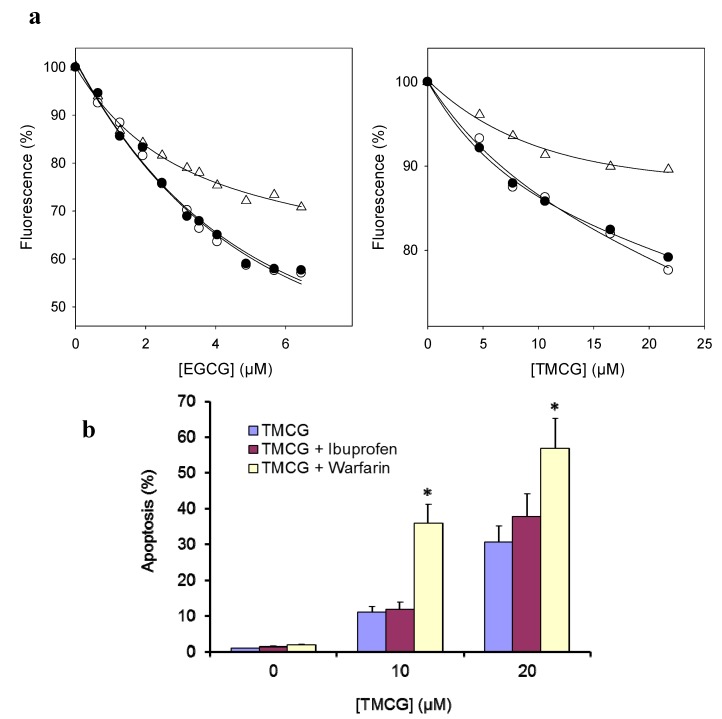
(**a**) Effect of site markers on the catechin-BSA complex (T = 25 °C; λ_ex_ = 280 nm). Effect of warfarin (△) or ibuprofen (●) on the quenching of BSA with EGCG and TMCG. (**b**) Effect of warfarin (10 µM) or ibuprofen (10 µM) on the pro-apoptotic activity of TMCG. * *p* < 0.05 with respect to cells treated with a single TMCG treatment.

Next, the site selective binding of both EGCG and TMCG to BSA was analyzed. The capability of serum albumins to bind to aromatic and heterocyclic compounds is largely dependent on the existence of two major binding regions, namely site-I and site-II [25]. Warfarin and ibuprofen are fluorescence probes whose primary binding sites to BSA are site-I and site-II, respectively. Competitive binding experiments [Figure 5(a) and Table 4] reveal that both EGCG and TMCG have higher specificity for site-I, at which they compete with warfarin. 

**Table 4:** Binding constants of catechins (EGCG and TMCG) to BSA in the presence of the site markers warfarin and ibuprofen at 25 °C.

Catechin	Site Marker	Binding constant (K_A_) (×10^6^ M^−1^)
EGCG	Blank	15.40
	Warfarin	0.13
	Ibuprofen	12.20
TMCG	Blank	0.19
	Warfarin	0.05
	Ibuprofen	0.11

Serum is commonly used as a supplement in cell culture media. It supports cell growth and product formation by providing a broad spectrum of macromolecules, attachment factors, nutrients, and hormone and growth factors. The most commonly used animal serum supplement is foetal bovine serum, FBS (alternatively called foetal calf serum—FCS). Because BSA is a major component of FBS, the *in vitro* activity of catechins on tumour cells might be affected by their interaction with BSA. The effect of albumin binding on the biological activity of polyphenols is unclear. Does the bound ligand have some biological activity or does the polyphenol have to be in the free form to be active? To answer this question and to determine whether BSA-catechin interactions increase or decrease catechin anti-tumour activity, the proapoptotic activity of TMCG on SK-MEL-28 melanoma cells was probed in the presence of warfarin or ibuprofen [Figure 5(b)]. As observed in this figure, warfarin or ibuprofen alone did not induce apoptosis in melanoma cells; however, warfarin, but not ibuprofen, significantly increased the proapoptotic activity of TMCG. These results indicate that serum albumins may have an important role, not only in the transportation and deposition of catechin-derived drugs in the blood but also on the impact on the distribution, free concentration, and metabolism of these drugs. Because the binding of catechins to BSA seems to control drug availability, the lower affinity of TMCG and TMECG for serum albumins, with respect to natural tea catechins, might contribute to the high *in vivo* anti-tumour activity of these methylated synthetic catechins. Therefore, the polyphenols characterised here might be useful to address this question in future *in vivo* studies.

## 3. Experimental

### 3.1. Synthesis

All reactions were carried out using solvents that were dried by routine procedures. ^1^H and ^13^C-NMR spectra were recorded on Bruker Avance 200 MHz, Bruker Avance 300 MHz, and Bruker Avance 400 MHz instruments. The following abbreviations are used to represent the multiplicity of the signals: s (singlet), bs (broad singlet), d (doublet), dd (double doublet), m (multiplet), and q (quaternary carbon atom). Chemical shifts are given with reference to the signal of (CH_3_)_4_Si in ^1^H and ^13^C-NMR spectra. Full assignments of ^1^H- and ^13^C NMR spectra were carried out by heteronuclear multiple quantum coherence (HMQC) experiment. Electrospray (ESI) mass spectra were recorded on Agilent 6220 TOF and Agilent VL spectrometers and FAB mass spectra were recorded on a Fisons AUTOSPEC 5000 VG spectrometer. Melting points were determined on a Kofler hot-plate melting point apparatus and are uncorrected. Compounds **1**–**4** were obtained using experimental procedures described elsewhere [16,19]. Compound **13b** was previously isolated by Gao *et al.* from the extract of the roots of *Lysidice rhodostega* and the spectral data of the synthesized compound are in complete agreement with their previously reported values [29].

#### 3.1.1. Synthesis of Acid Chlorides 7 and 9

*Benzyl 4-(benzyloxy)-3,5-dimethoxybenzoate* (**5**). A suspension of syringic acid (4.0 g, 20.1 mmol) and K_2_CO_3_ (27.9 g, 201 mmol) in acetone (150 mL) was refluxed under nitrogen for 20 min. Afterwards, benzyl bromide (9.6 mL, 80.7 mmol) was added dropwise and the mixture was refluxed for 7 h under nitrogen. On cooling, the reaction mixture was filtered and the solution was concentrated under vacuum to give **5** as a clear oil which was pure enough to be used in the next step without further purification. ^1^H-NMR (CDCl_3_, 400 MHz) δ 7.37–7.17 (m, 12H, Ph), 5.28 (s, 2H, CH_2_O), 5.00 (s, 2H, CH_2_O), 3.77 (s, 6H, 2 × OCH_3_). ^13^C-NMR (CDCl_3_, 100 MHz) δ 166.1 (q, COO), 153.2 (2 × q, Ar-OCH_3_), 141.1 (q, Ar-OBn), 137.7 (q, Ph-CH_2_O), 137.3 (q, Ph-CH_2_O), 128.4 (2 × CH, Ph), 128.3 (2 × CH, Ph), 128.2 (CH, Ph), 128.1 (2 × CH, Ph), 128.1 (2 × CH, Ph), 127.9 (CH, Ph), 125.2 (q, Ar-CO), 106.9 (2 × CH, Ar-H), 74.9 (CH_2_O), 66.7 (CH_2_O), 56.2 (2 × OCH_3_).

*4-(Benzyloxy)-3,5-dimethoxybenzoic Acid* (**6**). To a solution of benzyl 4-(benzyloxy)-3,5-dimethoxybenzoate **5** in methanol (100 mL) an aqueous solution of NaOH (5M, 25 mL) was added. The mixture was refluxed for 5 h and the resulting solution was concentrated under vacuum to remove the organic solvent. The residue was then dissolved in water and extracted with *n*-hexane (2 × 30 mL). The aqueous phase was acidified with HCl to pH 2 to give a white precipitate which was filtered, washed with chilled water and dried under reduced pressure (yield = 98%). MS (ESI) *m/z* (%) 287 (M^+^−1, 100). ^1^H-NMR (CDCl_3_, 200 MHz) δ 9.80 (bs, 1H, COOH), 7.42–7.17 (m, 7H, Ph), 5.04 (s, 2H, CH_2_O), 3.80 (s, 6H, 2×OCH_3_). ^13^C-NMR (CDCl_3_, 50 MHz) δ 171.7 (q, COOH), 153.2 (2 × q, Ar-OCH_3_), 141.7 (q, Ar-OBn), 137.2 (q, Ph-CH_2_O), 128.4 (2 × CH, Ph), 128.1 (2 × CH, Ph), 128.0 (CH, Ph), 124.2 (q, Ar-COO), 107.3 (2 × CH, Ar-H), 74.9 (CH_2_O), 56.1 (2 × OCH_3_).

*4-(Benzyloxy)-3,5-dimethoxybenzoyl Chloride* (**7**). To a solution of **6** (1 g, 3.4 mmol) in dry dichloromethane (20 mL) and under nitrogen, oxalyl chloride (0.6 mL, 6.9 mmol) was added dropwise. The reaction mixture was then stirred at room temperature for 8 h and then distilled under reduced pressure to give **7** as a white solid (yield = 97%). ^1^H-NMR (CDCl_3_, 200 MHz) δ 7.42–7.17 (m, 7H, Ph), 5.07 (s, 2H, CH_2_O), 3.82 (s, 6H, 2 × OCH_3_). ^13^C-NMR (CDCl_3_, 50 MHz) δ 167.5 (q, -COOCl), 153.3 (2 × q, Ar-OCH_3_), 143.2 (q, Ar-OBn), 136.9 (q, Ph-CH_2_O), 128.3 (2 × CH, Ph), 128.2 (2 × CH, Ph), 128.1 (CH, Ph), 127.8 (q, Ar-COO), 108.8 (2 × CH, Ar-H), 75.0 (CH_2_O), 56.3 (2 × OCH_3_).

*4-Acetoxy-3,5-dimethoxybenzoic Acid* (**8**). Syringic acid (1 g, 5.04 mmol) was added to acetic anhydride (7 mL) and the mixture was refluxed for 2 h. The hot reaction mixture was poured into ice-cold water and was left stirring for 1 h to form a white precipitate. The resulting precipitate was collected by filtration, washed with chilled water and dried under reduced pressure (yield = 83%). MS (ESI) *m/z* (%) 239 (M^+^−1, 100). ^1^H-NMR (CDCl_3_, 200 MHz) δ 10.89 (bs, 1H, COOH), 7.32 (s, 2H, Ar), 3.81 (s, 6H, 2 × OCH_3_), 2.28 (s, 3H, CH_3_COO). ^13^C-NMR (CDCl_3_, 50 MHz) δ 171.3 (q, -COOH), 168.1 (q, COO), 152.1 (2 × q, Ar-OCH_3_), 133.2 (q, Ar-COO), 127.1 (q, Ar-COOH), 106.7 (2 × CH, Ar-H), 56.2 (2 × OCH_3_), 20.3 (CH_3_COO).

*4-Acetoxy-3,5-dimethoxybenzoyl Chloride* (**9**). A mixture of 4-acetoxy-3,5-dimethoxybenzoic acid **8** (600 mg, 2.49 mmol) and thionylchloride (10 mL) was refluxed under nitrogen atmosphere for 2 h. On removing the excess of SOCl_2_ under vacuum a white solid was obtained in almost quantitative yield, which was used without further purification. ^1^H-NMR (CDCl_3_, 200 MHz) δ 7.31 (s, 2H, Ar), 3.82 (s, 6H, 2 × OCH_3_), 2.28 (s, 3H, CH_3_COO). ^13^C-NMR (CDCl_3_, 50 MHz) δ 167.7 (q, COO), 167.6 (q, COO), 152.3 (2 × q, Ar-OCH_3_), 134.6 (q, Ar-COO), 130.8 (q, Ar-COO), 108.0 (2 × CH, Ar), 56.4 (2 × OCH_3_), 20.3 (CH_3_COO).

#### 3.1.2. General Procedure for the Synthesis of Compounds **10b**, **10c**, and **11b**

A solution of the appropriate acid chloride **7** or **9** (3.91 mmol) in dry CH_2_Cl_2_ (5 mL) was added dropwise to a solution of the adequate derivative of catechin **1** or epicatechin **3** (1.3 g, 1.95 mmol) and dimethylaminopyridine (DMAP) (0.59 g, 4.87 mmol) in the same solvent (30 mL) and under nitrogen. The reaction mixture was stirred at room temperature for 18 h. Then, a solution of saturated sodium bicarbonate (40 mL) was added and the mixture was extracted with ethyl acetate (2 × 30 mL). The organic layers were washed with water (2 × 30 mL) and dried with anhydrous magnesium sulphate and the solvent removed under vacuum. The resulting yellow oils were chromatographed on a silica gel column using *n*-hexane/AcOEt/CH_2_Cl_2_ (6:6:2, v:v:v) as eluent. The solvent was removed under reduced pressure and the solids obtained were recrystallized from Et_2_O/*n*-hexane.

*5,7,3’,4’,4’’-penta-O-benzyl-3-O-(3’’,5’’-dimethoxybenzoyl)-catechin* (**10b**). Pale yellow solid (yield = 94%). Mp: 90–93 °C. MS (ESI) *m/z* (%) 921 (M^+^+1, 100). ^1^H-NMR (CDCl_3_, 400 MHz) δ 7.37–7.14 (m, 25H, Ph), 7.01 (s, 2H, H-2'' and H-6''), 6.96 (d, 1H, *^4^J* = 1.6 Hz, H-2'), 6.87 (dd, 1H, *^3^J* = 8.4 Hz, *^4^J* = 1.6 Hz, H-6'), 6.79 (d, 1H, *^3^J* = 8.4 Hz, H-5'), 6.19 (d, 1H, *^4^J* = 2.4 Hz, H-6), 6.18 (d, 1H, *^4^J* = 2.4 Hz, H-8), 5.37 (m, 1H, H-2), 5.02 (m, 3H, CH_2_O, H-3), 4.97 (s, 2H, CH_2_O), 4.95 (s, 2H, CH_2_O), 4.92 (s, 4H, 2 × CH_2_O), 3.68 (s, 6H, 2 × OCH_3_), 3.05 (m, 1H, Hgem, H-4), 2.76 (m, 1H, Hgem, H-4). ^13^C-NMR (CDCl_3_, 100 MHz) δ 165.2 (q, COO), 158.8 (q, Ar-O), 157.6 (q, Ar-O), 155.0 (q, Ar-O), 153.1 (2 × q, Ar-O), 149.0 (q, Ar-O), 148.9 (q, Ar-O), 141.1 (q, Ar-O), 137.3 (q, PhCH_2_), 137.0 (q, PhCH_2_), 136.9 (q, PhCH_2_), 136.8 (q, PhCH_2_), 136.7 (q, PhCH_2_), 131.0 (q, C-1’), 128.5 (CH, PhCH_2_), 128.4 (CH, PhCH_2_), 128.4 (CH, PhCH_2_), 128.3 (CH, PhCH_2_), 128.3 (CH, PhCH_2_), 128.1 (CH, PhCH_2_), 127.9 (CH, PhCH_2_), 127.9 (CH, PhCH_2_), 127.8 (CH, PhCH_2_), 127.7 (2 × CH, PhCH_2_), 127.4 (CH, PhCH_2_), 127.3 (CH, PhCH_2_), 127.1 (2 × CH, PhCH_2_), 125.0 (q, C-1''), 120.1 (CH, C-6'), 114.8 (CH, C-5'), 113.5 (CH, C-2'), 106.8 (CH, C-2'' and C-6''), 101.4 (q, C-4a), 94.3 (CH, C-6), 93.7 (CH, C-8), 78.5 (CH, C-2), 74.9 (CH_2_, CH_2_Ph), 71.3 (CH_2_, CH_2_Ph), 71.1 (CH_2_, CH_2_Ph), 70.1 (CH, C-3), 70.1 (CH_2_, CH_2_Ph), 69.9 (CH_2_, CH_2_Ph), 56.1 (OCH_3_), 24.6 (CH_2_, C-4).

*4’’-O-acetyl-5,7,3’,4’-tetra-O-benzyl-3-O-(3’’,5’’-dimethoxybenzoyl)-catechin* (**10c**). White solid (yield = 75%). Mp: 92–96 °C. MS (FAB^+^) *m/z* (%) 873 (M^+^+1, 10). ^1^H-NMR (CDCl_3_, 400 MHz) δ 7.33–7.19 (m, 25H, Ph), 7.04 (s, 2H, H-2'' and H-6''), 6.96 (d, 1H, *^4^J* = 1.8 Hz, H-2'), 6.87 (dd, 1H, *^3^J* = 8.2 Hz, *^4^J* = 1.8 Hz, H-6’), 6.79 (d, 1H, *^3^J* = 8.2 Hz, H-5'), 6.20 (d, 1H, *^4^J* = 2.4 Hz, H-6), 6.18 (d, 1H, *^4^J* = 2.4 Hz, H-8), 5.39 (m, 1H, H-3), 5.04 (s, 3H, CH_2_O and H-2), 4.98 (s, 2H, CH_2_O), 4.94 (s, 2H, CH_2_O), 4.93 (s, 2H, CH_2_O), 3.69 (s, 6H, 2 × OCH_3_), 3.04 (m, 1H, Hgem, H-4), 2.76 (m, 1H, Hgem, H-4), 2.26 (s, 3H, CH_3_COO). ^13^C-NMR (CDCl_3_, 100 MHz) δ 168.1 (q, COOCH_3_), 164.9 (q, COO), 158.8 (q, Ar-O), 157.6 (q, Ar-O), 154.9 (2 × q, Ar-O), 151.9 (q, Ar-O), 149.1 (q, Ar-O), 148.9 (q, Ar-O), 137.0 (q, PhCH_2_), 136.9 (q, PhCH_2_), 136.8 (q, PhCH_2_), 136.7 (q, PhCH_2_), 132.5 (q, Ar-O), 130.9 (q, C-1'), 128.6 (CH, PhCH_2_), 128.5 (CH, PhCH_2_), 128.4 (2 × CH, PhCH_2_), 128.0 (CH, PhCH_2_), 127.9 (2 × CH, PhCH_2_), 127.7 (CH, PhCH_2_), 127.5 (CH, PhCH_2_), 127.4 (CH, PhCH_2_), 127.1 (2 × CH and q, PhCH_2_ and C-1''), 120.1 (CH, C-6'), 114.7 (CH, C-5'), 113.5 (CH, C-2'), 106.2 (CH, C-2'' and C-6''), 101.3 (q, C-4a), 94.3 (CH, C-6), 93.7 (CH, C-8), 78.4 (CH, C-2), 71.4 (CH_2_, CH_2_Ph), 71.1 (CH_2_, CH_2_Ph), 70.3 (CH, C-3), 70.0 (CH_2_, CH_2_Ph), 69.9 (CH_2_, CH_2_Ph), 56.2 (OCH_3_), 24.5 (CH_2_, C-4), 20.3 (CH_3_COO).

*5,7,3’,4’,4’’-penta-O-benzyl-3-O-(3’’,5’’-dimethoxybenzoyl)-epicatechin* (**11b)**. White solid (yield = 97%). MS (ESI) *m/z* (%) 920.9 (M^+^+1, 100). ^1^H-NMR (CDCl_3_, 400 MHz) δ 7.32–7.20 (m, 25H, Ph), 7.07 (s, 2H, H-2'' and H-6''), 7.05 (d, 1H, *^4^J* = 1.8 Hz, H-2'), 6.92 (dd, 1H, *^3^J* = 8.2 Hz, *^4^J* = 1.8 Hz, H-6’), 6.80 (d, 1H, *^3^J* = 8.2 Hz, H-5'), 6.22 (d, 1H, *^4^J* = 2.2 Hz, H-6), 6.20 (d, 1H, *^4^J* = 2.2 Hz, H-8), 5.01 (s, 4H, 2 × CH_2_O), 4.93 (s, 2H, CH_2_O), 4.91 (s, 2H, CH_2_O), 4.89 (s, 2H, CH_2_O), 4.90 (d, 1H, *^3^J* = 11.7 Hz, H-2), 4.77 (d, 1H, *^3^J* = 11.7 Hz, H-3), 3.66 (s, 6H, 2 × OCH_3_), 3.01 (m, 2H, Hgem, H-4). ^13^C-NMR (CDCl_3_, 100 MHz) δ 165.2 (q, COO), 158.7 (q, Ar-O), 157.8 (q, Ar-O), 155.5 (q, Ar-O), 153.0 (2 × q, Ar-O), 148.8 (2 × q, Ar-O), 141.0 (q, Ar-O), 137.2 (q, PhCH_2_), 137.0 (q, PhCH_2_), 136.9 (q, PhCH_2_), 136.7 (2 × q, PhCH_2_), 131.0 (q, C-1’), 128.5 (CH, PhCH_2_), 128.4 (CH, PhCH_2_), 128.3 (CH, PhCH_2_), 128.3 (CH, PhCH_2_), 128.2 (CH, PhCH_2_), 128.1 (CH, PhCH_2_), 127.9 (CH, PhCH_2_), 127.8 (CH, PhCH_2_), 127.8 (CH, PhCH_2_), 127.7 (CH, PhCH_2_), 127.7 (CH, PhCH_2_), 127.4 (CH, PhCH_2_), 127.2 (CH, PhCH_2_), 127.1 (CH, PhCH_2_), 127.1 (CH, PhCH_2_), 125.1 (q, C-1''), 119.9 (CH, C-6'), 114.4 (CH, C-5'), 113.8 (CH, C-2'), 106.9 (CH, C-2 and C-6''), 100.7 (q, C-4a), 94.4 (CH, C-6), 93.7 (CH, C-8), 77.3 (CH, C-2), 74.8 (CH_2_, CH_2_Ph), 71.3 (CH_2_, CH_2_Ph), 71.1 (CH_2_, CH_2_Ph), 70.0 (CH_2_, CH_2_Ph), 69.8 (CH_2_, CH_2_Ph), 68.7 (CH, C-3), 56.1 (OCH_3_), 25.8 (CH_2_, C-4).

#### 3.1.3. General Procedure for the Synthesis of Compounds **12b**, **12c**, and **13b**

Under normal pressure, a solution of the corresponding benzyl eter derivative **10b**, **10c **or **11b** (1.5 mmol) and 10% Pd/C (0.05 g of palladium, 0.47 mmol) in THF/MeOH (3:1) (40 mL) was treated with molecular hydrogen. The solution was stirred for 15 h at room temperature and then filtered on a cellite pad which, afterwards, was washed with CH_2_Cl_2_/MeOH (9:1) (250 mL). The solvent was removed under vacuum and the resulting white solids were recrystallized from Et_2_O. 

*3-O-(4-hydroxy-3,5-dimethoxybenzoyl)-catechin* (**12b**). White solid (yield = 80%). Mp: 110–114 °C. MS (ESI) *m/z* (%) 469 (M^+^−1, 100). ^1^H-NMR (acetone-d_6_, 400 MHz) δ 8.40 (bs, 1H, OH), 8.17 (bs, 1H, OH), 8.05 (bs, 1H, OH), 7.99 (bs, 1H, OH), 7.97 (bs, 1H, OH), 7.15 (s, 2H, H-2'' and H-6''), 6.99 (d, 1H, *^4^J* = 1.8 Hz, H-2'), 6.87 (dd, 1H, *^3^J* = 8.2 Hz, *^4^J* = 1.8 Hz, H-6'), 6.80 (d, 1H, *^3^J* = 8.2 Hz, H-5’), 6.07 (d, 1H, *^4^J* = 2.2 Hz, H-6), 5.96 (d, 1H, *^4^J* = 2.2 Hz, H-8), 5.25 (m, 1H, H-3), 5.05 (d, 1H, *J* = 7.6 Hz, H-2), 3.81 (s, 6H, 2 × OCH_3_), 3.12 (m, 1H, Hgem, H-4), 2.73 (m, 1H, Hgem, H-4). ^13^C-NMR (acetone-d_6_, 100 MHz) δ 165.5 (q, COO), 157.7 (q, Ar-O), 156.9 (q, Ar-O), 156.2 (q, Ar-O), 147.9 (2 × q, Ar-O), 145.5 (2 × q, Ar-O), 141.3 (q, Ar-O), 130.8 (q, C-1'), 120.8 (q, C-1''), 119.1 (CH, C-6'), 115.5 (CH, C-5'), 114.4 (CH, C-2'), 107.5 (CH, C-2'' and C-6''), 99.1 (q, C-4a), 96.1 (CH, C-6), 95.1 (CH, C-8), 79.1 (CH, C-2), 71.1 (CH, C-3), 56.2 (CH_3_O), 25.6 (CH_2_, C-4). 

*3-O-(4-acetoxy-3,5-dimethoxybenzoyl)-catechin*(**12c**). White solid (yield = 95%). Mp: 118–122 °C. MS (ESI) *m/z* (%) 512 (M^+^−1, 100). ^1^H-NMR (acetone-d_6_, 400 MHz) δ 8.41 (bs, 1H, OH), 8.16 (bs, 1H, OH), 7.97 (bs, 1H, OH), 7.96 (bs, 1H, OH), 7.97 (bs, 1H, OH), 7.17 (s, 2H, H-2’’ and H-6’’), 7.00 (d, 1H, *^4^J* = 2 Hz, H-2’), 6.88 (dd, 1H, *^3^J* = 8 Hz, *^4^J* = 2 Hz, H-6’), 6.81 (d, 1H, *^3^J* = 8 Hz, H-5’), 6.07 (d, 1H, *^4^J* = 2 Hz, H-6), 5.96 (d, 1H, *^4^J* = 2 Hz, H-8), 5.29 (m, 1H, H-3), 5.05 (d, 1H, *J* = 7.6 Hz, H-2), 3.81 (s, 6H, 2×OCH_3_), 3.15 (m, 1H, Hgem, H-4), 2.76 (m, 1H, Hgem, H-4), 2.22 (s, 3H, CH_3_COO-). ^13^C-NMR (acetone-d_6_, 100 MHz) δ 167.9 (q, -COOCH_3_), 165.2 (q, COO), 158.0 (q, Ar-O), 157.0 (q, Ar-O), 156.4 (q, Ar-O), 153.0 (2 × q, Ar-O), 145.8 (q, Ar-O), 145.7 (q, Ar-O), 133.5 (q, Ar-O), 130.9 (q, C-1'), 128.9 (q, C-1''), 119.3 (CH, C-6'), 115.7 (CH, C-5'), 114.6 (CH, C-2'), 106.6 (CH, C-2'' and C-6''), 99.1 (q, C-4a), 96.2 (CH, C-6), 95.3 (CH, C-8), 79.1 (CH, C-2), 71.8 (CH, C-3), 56.4 (CH_3_O), 25.7 (CH_2_, C-4), 19.9 (CH_3_COO). 

*3-O-(4-hydroxy-3,5-dimethoxybenzoyl)-epicatechin* (**13b**). White solid (yield = 46%). Mp: 120–123 °C. MS (ESI) *m/z* (%) 469 (M^+^−1, 100). ^1^H-NMR (acetone-d_6_, 400 MHz) δ 8.32 (bs, 1H, OH), 8.12 (bs, 1H, OH), 8.01 (bs, 1H, OH), 7.96 (bs, 1H, OH), 7.82 (bs, 1H, OH), 7.17 (s, 2H, H-2'' and H-6''), 7.13 (d, 1H, *^4^J* = 2 Hz, H-2'), 6.91 (dd, 1H, *^3^J* = 8 Hz, *^4^J* = 2 Hz, H-6’), 6.80 (d, 1H, *^3^J* = 8 Hz, H-5’), 6.06 (d, 1H, *^4^J* = 2.4 Hz, H-6), 6.04 (d, 1H, *^4^J* = 2.4 Hz, H-8), 5.49 (m, 1H, H-3), 5.20 (s, 1H, H-2), 3.82 (s, 6H, 2 × OCH_3_), 3.08 (m, 1H, Hgem, H-4), 3.00 (m, 1H, Hgem, H-4). ^13^C-NMR (acetone-d_6_, 100 MHz) δ 166.0 (q, COO), 157.8 (q, Ar-O), 157.7 (q, Ar-O), 156.9 (q, Ar-O), 148.2 (q, Ar-O), 145.5 (q, Ar-O), 145.4 (q, Ar-O), 141.5 (q, Ar-O), 131.4 (q, C-1'), 121.2 (q, C-1''), 118.8 (CH, C-6'), 115.6 (CH, C-5'), 114.7 (CH, C-2’), 107.6 (CH, C-2'' and C-6''), 98.7 (q, C-4a), 96.4 (CH, C-6), 95.5 (CH, C-8), 77.7 (CH, C-2), 70.2 (CH, C-3), 56.5 (CH_3_O), 26.0 (CH_2_, C-4).

#### 3.1.4. Synthesis of Compound **12d**

*5,7,3’,4’,4’’-penta-O-acetyl-3-O-(3,5-dimethoxybenzoyl)-catechin* (**12d**). A mixture of compound **12b** (50 mg, 0.106 mmol), pyridine (2 mL) and acetic anhydride (3 mL) was stirred for 16 h at 40 °C in an oil bath. The reaction mixture was then poured into ice-cold water (60 mL) with vigorous stirring and was left stirring for 40 min to form a white precipitate which was collected by filtration, washed with chilled water and dried under reduced pressure to yield **12d **(63%). Mp: 88–93 °C. MS (ESI) *m/z* (%) 703 (M^+^+ 23 (Na^+^), 100). ^1^H-NMR (acetone-d_6_, 300 MHz) δ 7.48 (dd, 1H, *^3^J* = 8.1 Hz, *^4^J* = 1.8 Hz, H-6'), 7.45 (d, 1H, *^4^J* = 1.8 Hz, H-2'), 7.28 (d, 1H, *^3^J* = 8.1 Hz, H-5'), 7.19 (s, 2H, H-2’’ and H-6''), 6.68 (d, 1H, *^4^J* = 2.1 Hz, H-6), 6.61 (d, 1H, *^4^J* = 2.1 Hz, H-8), 5.46 (m, 1H, H-3), 5.42 (d, 1H, *J* = 6.9 Hz, H-2), 3.80 (s, 6H, 2 × OCH_3_), 3.11 (m, 1H, Hgem, H-4), 2.87 (m, 1H, Hgem, H-4), 2.26 (s, 3H, CH_3_COO), 2.23 (s, 12H, 4 × CH_3_COO). ^13^C-NMR (acetone-d_6_, 75 MHz) δ 169.1 (q, COO), 169.8 (q, COO), 168.3 (2 × q, COO), 167.9 (q, COO), 164.8 (q, COO), 155.3 (q, Ar-O), 153.0 (2 × q, Ar-O), 150.9 (q, Ar-O), 150.5 (q, Ar-O), 143.3 (q, Ar-O), 143.3 (q, Ar-O), 137.0 (q, Ar-O), 133.6 (q, C-1'), 128.2 (q, C-1''), 125.4 (CH, C-6'), 124.4 (CH, C-5'), 122.7 (CH, C-2'), 111.4 (q, C-4a), 109.8 (CH, C-8), 108.1 (CH, C-6), 106.7 (CH, C-2'' and C-6''), 78.6 (CH, C-2), 70.2 (CH, C-3), 56.4 (CH_3_, CH_3_O), 25.3 (CH_2_, C-4), 20.6 (CH_3_COO), 20.4 (CH_3_COO), 20.2 (CH_3_COO), 20.2 (CH_3_COO), 19.9 (CH_3_COO).

### 3.2. Biological Activities

#### 3.2.1. Materials

Highly purified tea polyphenols EGCG (> 95%), ECG (> 98%), and GC (> 98%) were purchased from Sigma-Aldrich (Madrid, Spain). Human DHFR was expressed in *Bombyx mori* chrysalides and purified by MTX-affinity chromatography [30]. The enzyme concentration was determined by MTX titration of the enzyme fluorescence [31]. BSA, HSA, warfarin, and ibuprofen were also purchased from Sigma-Aldrich. 

#### 3.2.2. Cell lines, Proliferation, and Apoptosis Assays

The human melanoma cell line SK-MEL-28 was obtained from American Type Culture Collection (ATCC, Manassas, VA, USA) and was maintained in Eagles’s Minimum Essential Medium (EMEM) supplemented with 10% (v/v) FBS and antibiotics at 37 °C in a humidified atmosphere containing 7.5% CO_2_. Cell viability was evaluated using the MTT cell proliferation assay. Cells were evaluated for apoptosis using a cell death detection ELISA (Cell Death Detection ELISAPLUS, Roche Diagnostics, Barcelona, Spain). The principle of this test is the detection of mono- and oligonucleosomes in the cytoplasmic fractions of cell lysates using biotinylated anti-histone and peroxidase-coupled anti-DNA antibodies. The amount of nucleosomes is photometrically quantified at 405 nm by the peroxidase activity retained in the immunocomplexes. Apoptosis was defined as the specific enrichment of mono- and oligonucleosomes in the cytoplasm and was calculated by dividing the absorbance of treated samples with the absorbance of untreated samples after correcting for the number of cells. The induction of apoptosis in each melanoma cell line after a 7 h treatment with 2 μM staurosporin (100% apoptotic cells) was used to calculate the number of apoptotic cells. For these assays, cells were plated in a 96-well plate at a density of 1000–2000 cells/well. Compounds were added once at the beginning of the experiments.

#### 3.2.3. DHFR Binding Studies

The dissociation constants for the binding of polyphenols to free recombinant human DHFR (rHDHFR) were determined by fluorescence titration in an automatic-scanning FluoroMax-3 (Jobin Ybon, Horiba, Edison, NJ, USA) spectrofluorometer with 1.0 cm light path cells and a 150 W mercury-xenon light source. The formation of a binary complex between the enzyme (2.5 × 10^−7^ M) and the ligand (from 0 to 40 µM) was followed by measuring the quenching of the tryptophan fluorescence of the enzyme upon the addition of microliter volumes of a concentrated stock solution of ligand. Experiments were performed at pH 7.5 in a buffer containing 2-(N-morpholino)ethanesulfonic acid (Mes, 0.025 M), sodium acetate (0.025 M), tris(hydroxymethyl)aminomethane (Tris 0.05 M), and NaCl (0.1 M). The temperature was controlled at 25 °C using a Haake D1G circulating bath with a heater and cooler. Fluorescence emission spectra were recorded when the human DHFR fluorescence was excited at 290 nm, and titrations were performed as described elsewhere [11]. 

#### 3.2.4. BSA Binding Studies

Fluorescence measurements were made on an automatic scanning Perkin-Elmer LS50B spectrofluorometer with 1.0 cm light path cells equipped with a 150 W xenon (XBO) light source. In all experiments, the fluorescence of BSA (5 × 10^−6^ M) in 50 mM phosphate buffer of pH 7.5 was followed by excitation at 280 nm. The binding affinities of EGCG and TMCG with BSA in terms of binding constants were determined using the following equation [25]:

*log (F_0_−F)/F = log K_A_ + n log [Q]* (1)

where *K_A_* is the binding constant, *n* is the number of binding sites, *F_0_* is the fluorescence intensity of free BSA and *F* is the consequent fluorescence upon the addition of catechins (*Q*). A plot of *log (F_0_-F)/F* against *log [Q]* was used to determine the values of *K_A_* and *n* from the intercept and the slope, respectively.

To identify the binding sites of EGCG and TMCG in BSA, the displacements of warfarin and ibuprofen (known to bind at site I and site II, respectively) were followed fluorometrically. Equimolar solutions of BSA and warfarin (5 × 10^−6^ M each) were mixed for 1 h and titrated with EGCG and TMCG in separate experiments. Similar solutions containing equimolar ibuprofen and BSA were prepared and separately titrated with EGCG and TMCG to identify the binding sites of EGCG and TMCG with BSA. Titration was followed fluorometrically using an excitation wavelength of 280 nm. 

#### 3.2.5. HSA Binding Studies

Binding of TMECG, EGCG, and EC to HSA was determined as specified for the BSA binding studies.

#### 3.2.6. Spectrophotometric Assays

Ultraviolet-visible absorption spectra of catechins at different pHs were recorded on a UV-Vis Perkin-Elmer Lambda-2 spectrophotometer with a spectral bandwidth of 1 nm at a scan speed of 60 nm s^−1^. The experiments were performed in the same buffer as described for DHFR binding studies, and the ionic strength remained constant at an optimum value of 0.15 over the pH range that was used. The pH of the reaction was measured before and after the experiment.

#### 3.2.7. Statistical Analysis

In all experiments, the mean ± standard deviation (SD) values for three to five determinations in triplicate were calculated. Statistically significant differences were evaluated using the Student’s t-test. Differences were considered statistically significant at *p* < 0.05.

## 4. Conclusions

Recent studies have indicated that natural tea catechins or related synthetic catechins are good inhibitors of DHFR. In this study, we show that polyphenols with catechin configuration are better DHFR inhibitors than those with epicatechin configuration. However, in addition to their DHFR inhibiting characteristics, other aspects should be considered for the design and syntheses of these types of inhibitors. For example, although an OH group in *para* position of ring D seems to be an important determinant that favours DHFR-catechin interaction, the pH-dependent ionization of this hydroxyl group impedes drug transportation through the cell membrane, which highly reduces the biological activity of these compounds. The interaction of catechins with albumin seems to be another important factor that might modulate the *in vivo* activity of these drugs. Although natural galloylated catechins, such as EGCG or ECG, are better inhibitors of DHFR, the high affinity of these catechins for serum albumin might reduce their bioavailability. In summary, we found that two synthetic methylated catechins, TMECG and TMCG, presented an ideal balance between DHFR inhibition and other factors that improve their cell and body bioavailability, such as high hydrophobicity and low interaction with serum albumins. These results demonstrate the high *in vitro* and *in vivo* anti-tumour activity of these two catechins on different experimental models of cancer [32,33]. These observations, together with their low toxicity on non-cancer cells [33,34], indicate that these drugs might have a great potential for their application in cancer therapies. Finally, the observation that the displacement of the equilibrium of the catechin-BSA complex using a competitive inhibitor of the albumin binding site-I, such as warfarin, highly increases the anti-tumour activity of TMCG, may be useful for the design and formulation of catechin-containing therapies. The binding of sulphonamide drugs to serum albumin has long been considered important for their chemotherapeutic values, such as *in vivo* antibacterial activity and the duration of action [35]. In this respect, the effect of these catechins on the pharmacokinetics and pharmacodynamics of warfarin, a commonly used drug for preventing intravascular coagulation, should be determined in order to evaluate the potential side-effects and clinical consequence of these findings.

## Supplementary Materials

Supplementary materials containing ^1^H and ^13^C NMR and mass spectrometry spectra of compounds **10b**, **10c**, **11b**, **12b**–**d**, and **13b** can be accessed at: http://www.mdpi.com/1420-3049/18/7/8319/s1.
