# Supplementary material for: Factors Influencing the Antifolate Activity of Synthetic Tea-Derived Catechins
Source: Molecules. 2013 Jul 16;18(7):8319–41. doi: 10.3390/molecules18078319 (PMC6270263; doi:10.3390/molecules18078319)

Supplementary Materials

Figure S1. Spectra of 5,7,3',4',4''-penta-*O*-benzyl-3-*O*-(3'',5''-dimethoxybenzoyl)-catechin (**10b**).

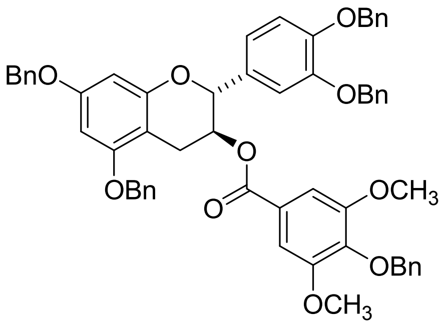

^{1}H-RMN spectrum of **10b**

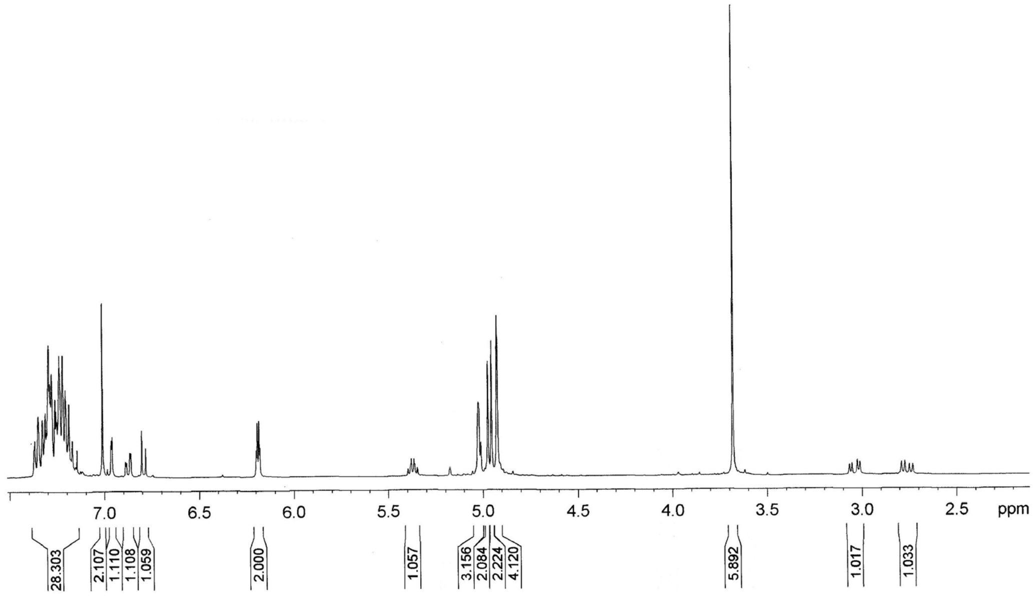

^{13}C-RMN spectrum of **10b**

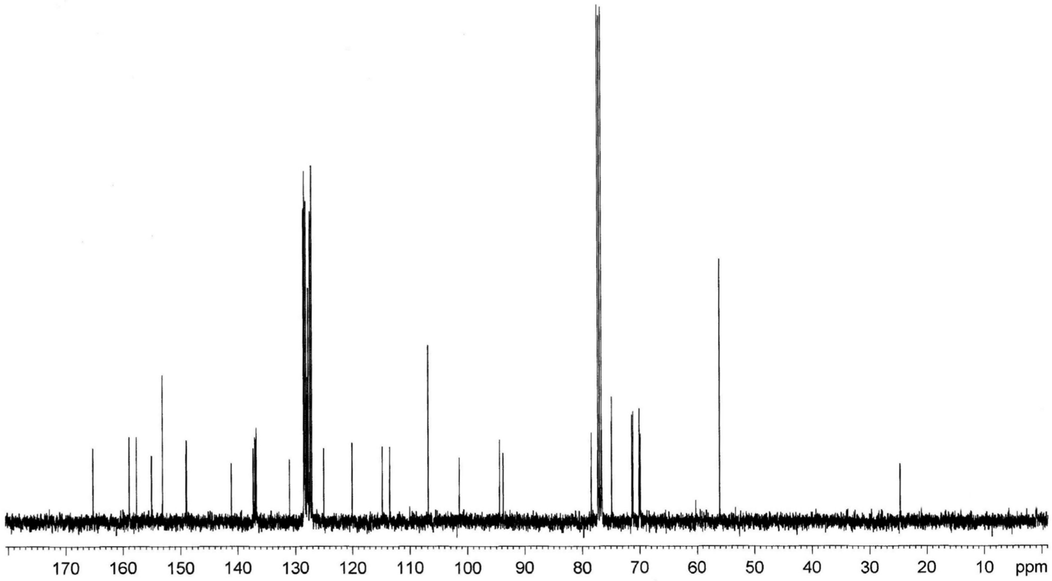

ESI-MS spectrum of **10b**

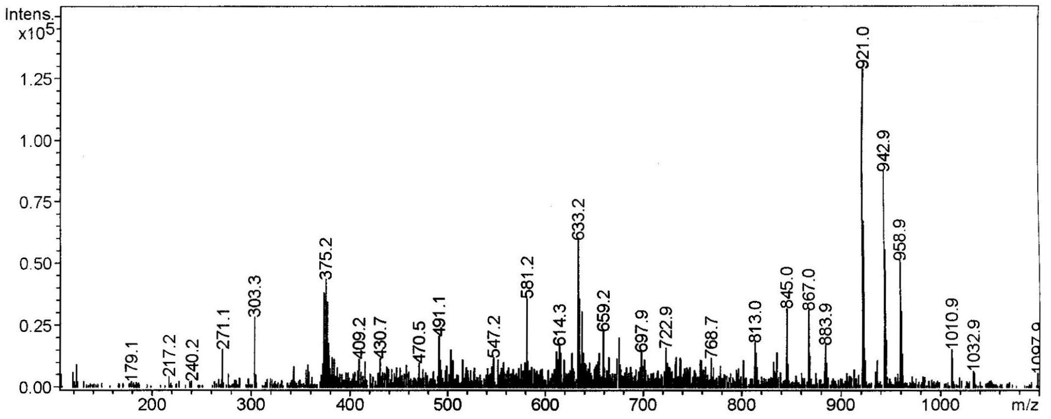

Figure S2. Spectra of 4''-*O*-acetyl-5,7,3',4'-tetra-*O*-benzyl-3-O-(3'',5''-dimethoxybenzoyl)-catechin (**10c**).

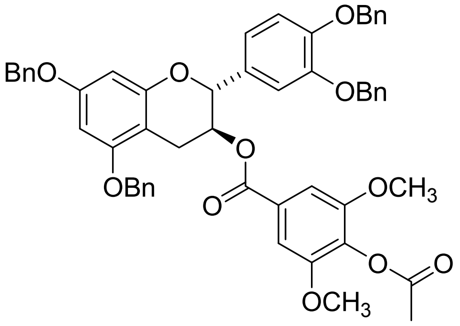

^{1}H-RMN spectrum of **10c**

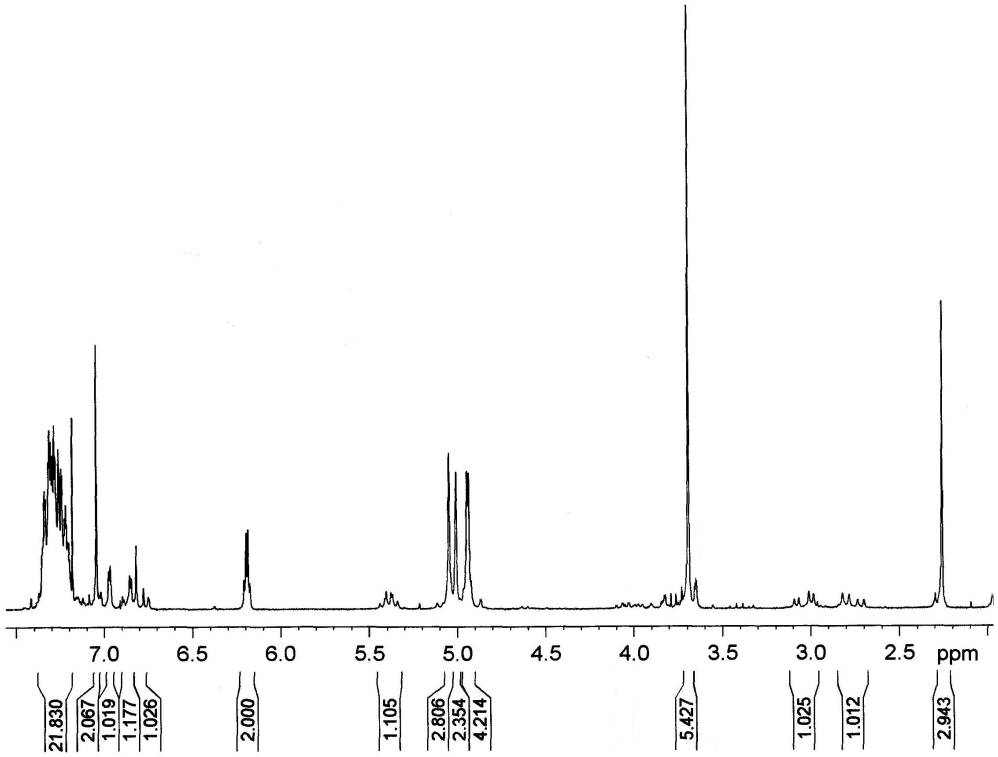

^{13}**C-RMN** spectrum of **10c**

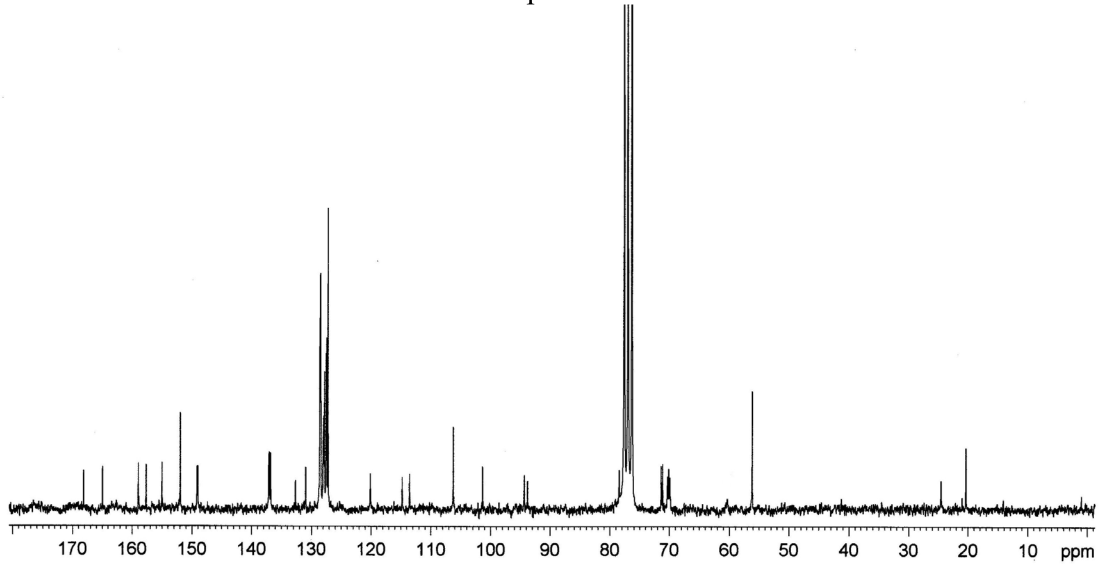

HMQC-RMN spectrum of **10c**

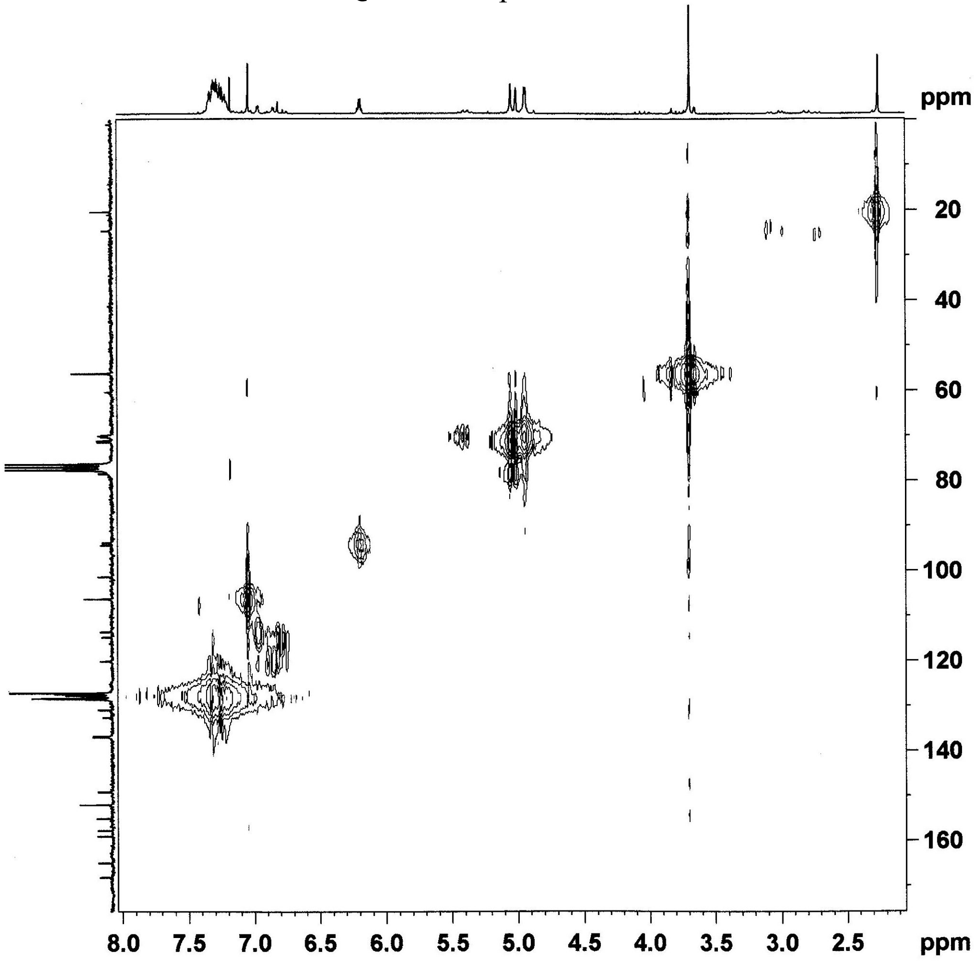

FAB-MS spectrum of **10c**

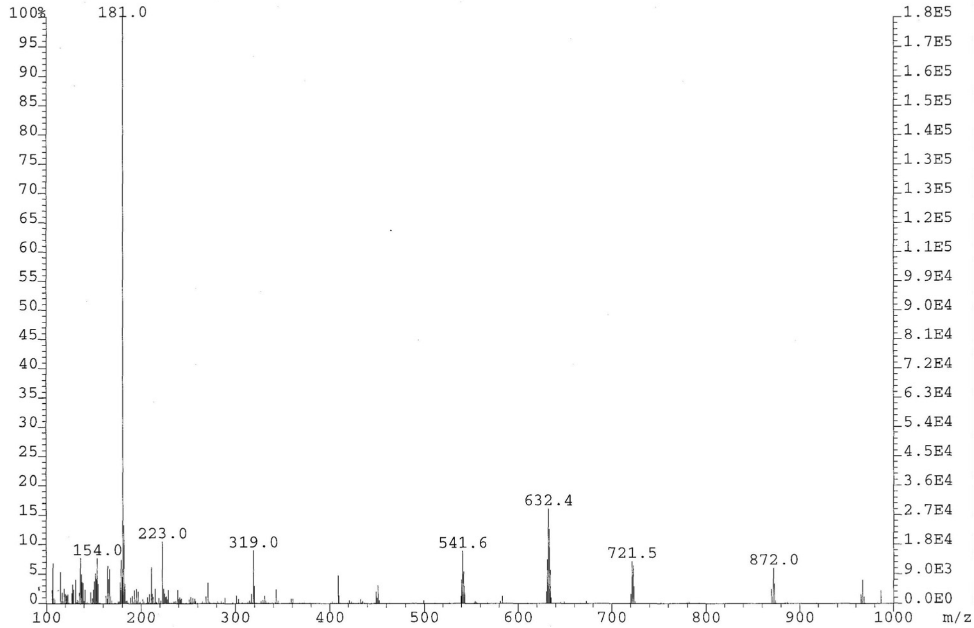

Figure S3. 5,7,3',4',4''-penta-*O*-benzyl-3-*O*-(3'',5''-dimethoxybenzoyl)-epicatechin (**11b**).

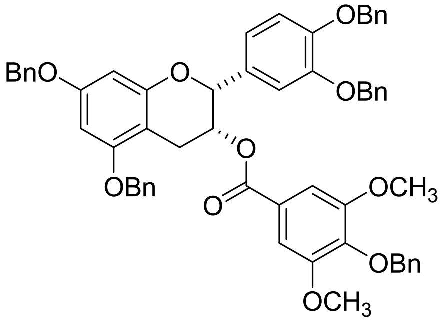

^{1}H-RMN spectrum of **11b**

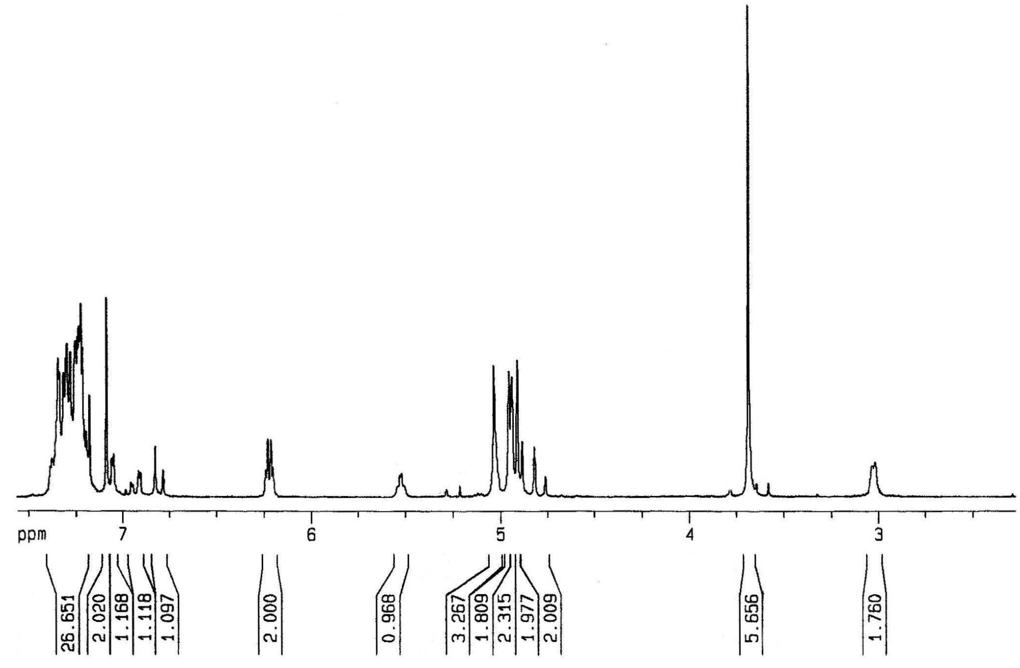

^{13}C-RMN spectrum of **11b**

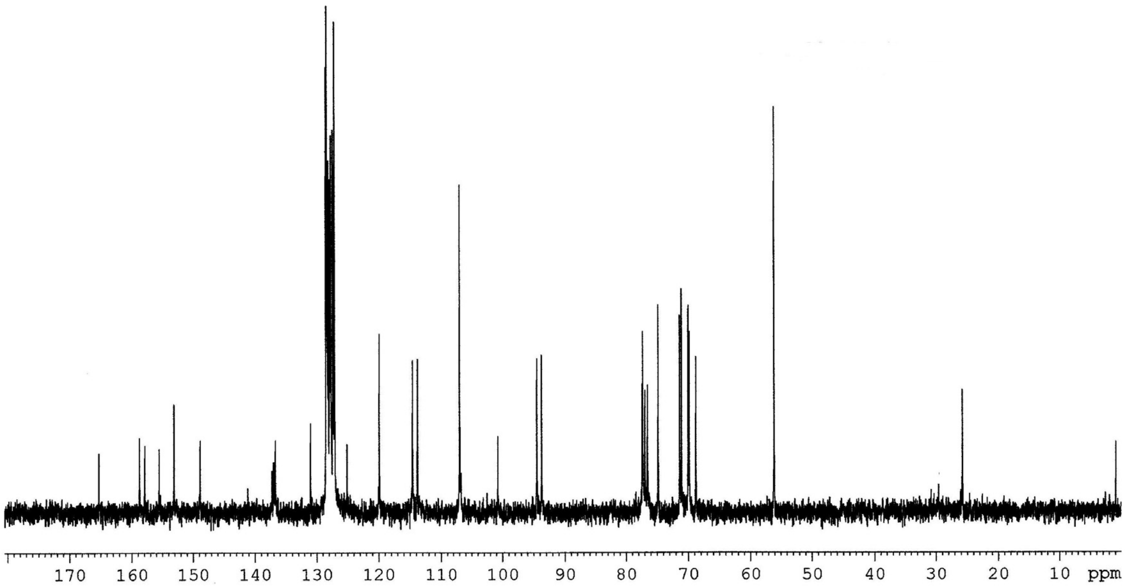

ESI-MS spectrum of **11b**

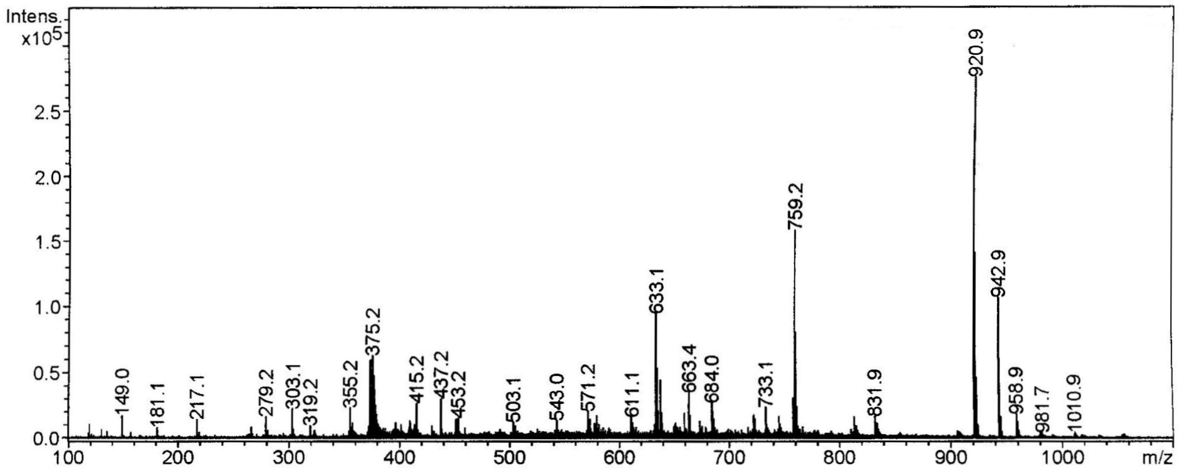

Figure S4. Spectra of 3-*O*-(4-hydroxy-3,5-dimethoxybenzoyl)-catechin (**12b**).

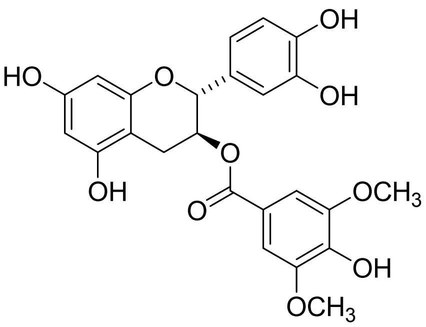

^{1}H-RMN spectrum of **12b**

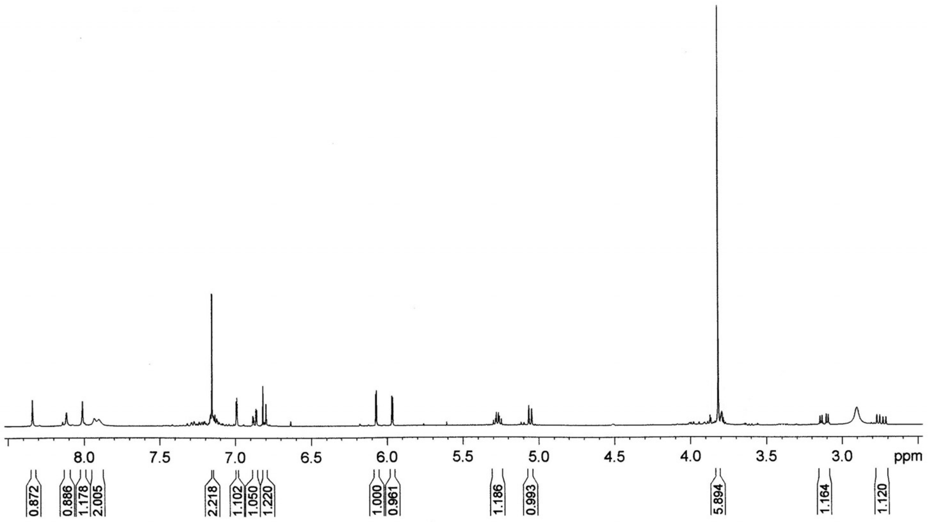

^{13}C-RMN spectrum of **12b**

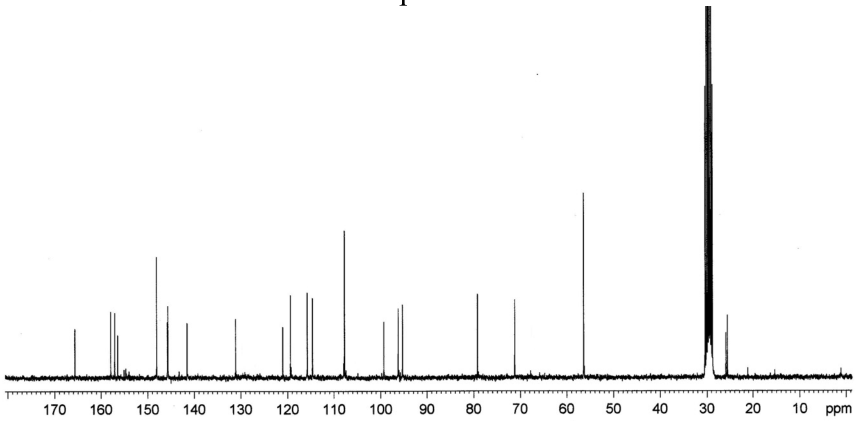

HMQC-RMN spectrum of **12b**

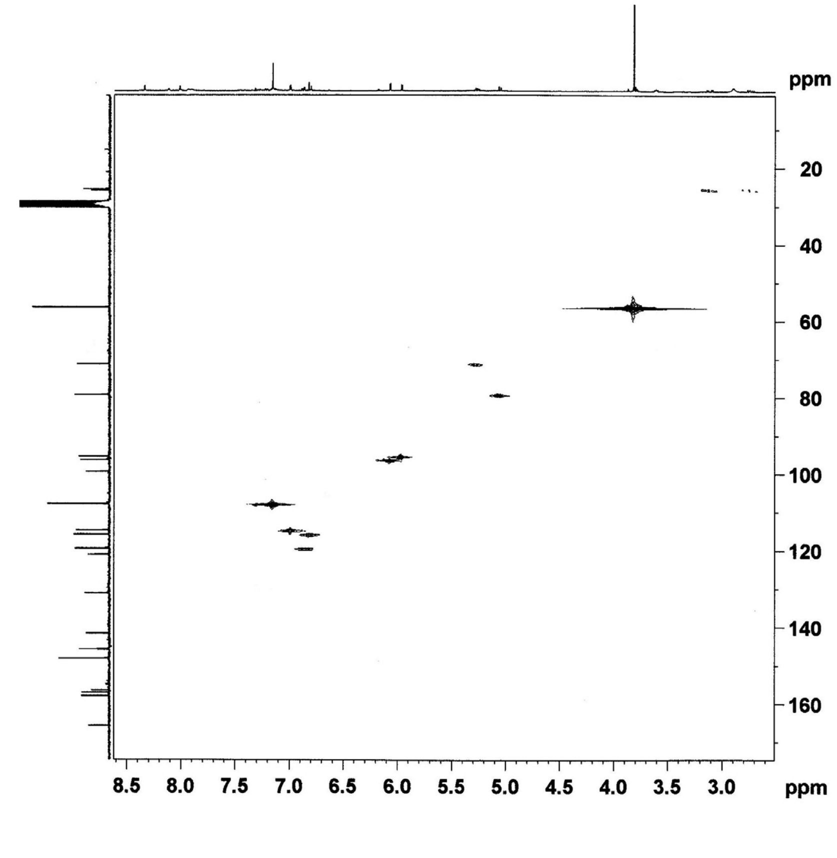

ESI-MS spectrum of **12b**

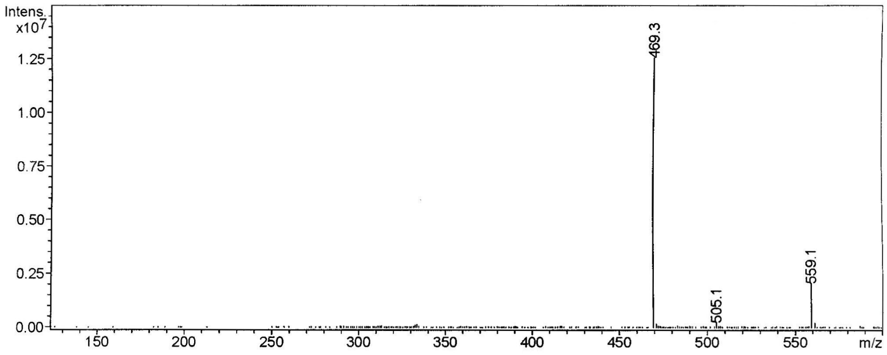

Figure S5. Spectra of 3-*O*-(4-acetoxy-3,5-dimethoxybenzoyl)-catechin (**12c**).

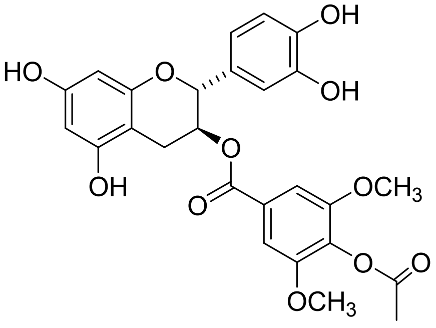

[1]**H-RMN** spectrum of **12c**

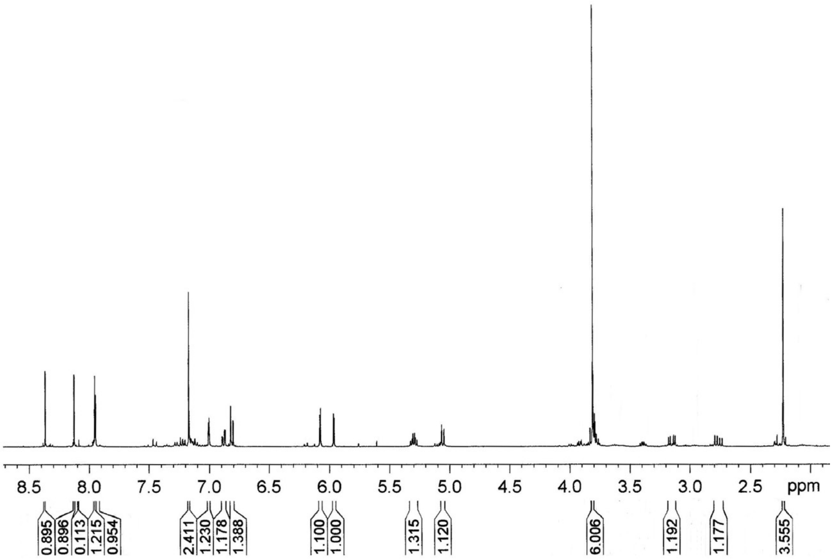

[13]**C-RMN** spectrum of **12c**

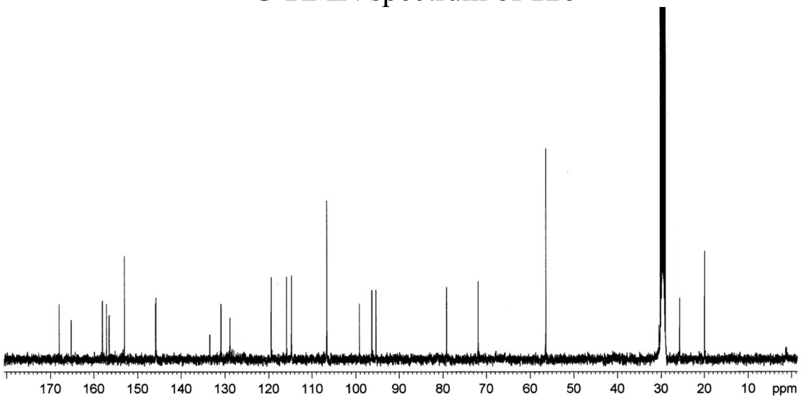

HMQC-RMN spectrum of **12c**

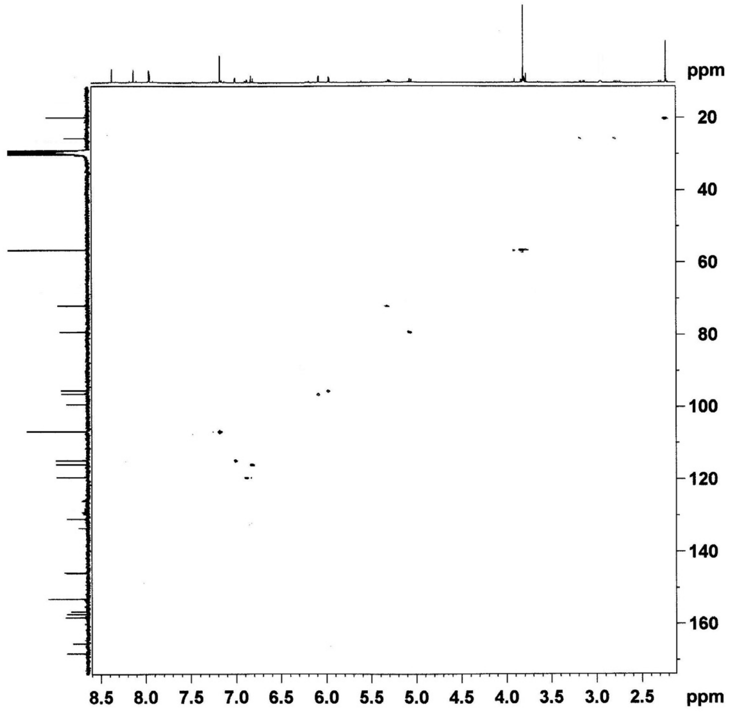

ESI-MS spectrum of **12c**

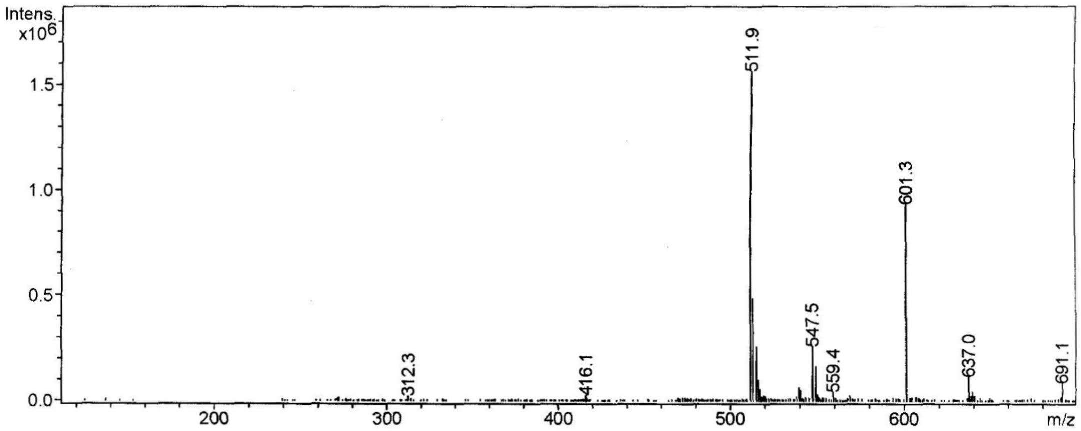

Figure S6. Spectra of 5,7,3',4',4''-penta-*O*-acetyl-3-*O*-(3,5-dimethoxybenzoyl)-catechin (**12d**).

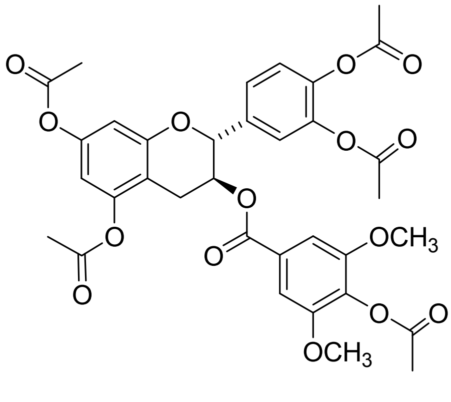

^{1}H-RMN spectrum of **12d**

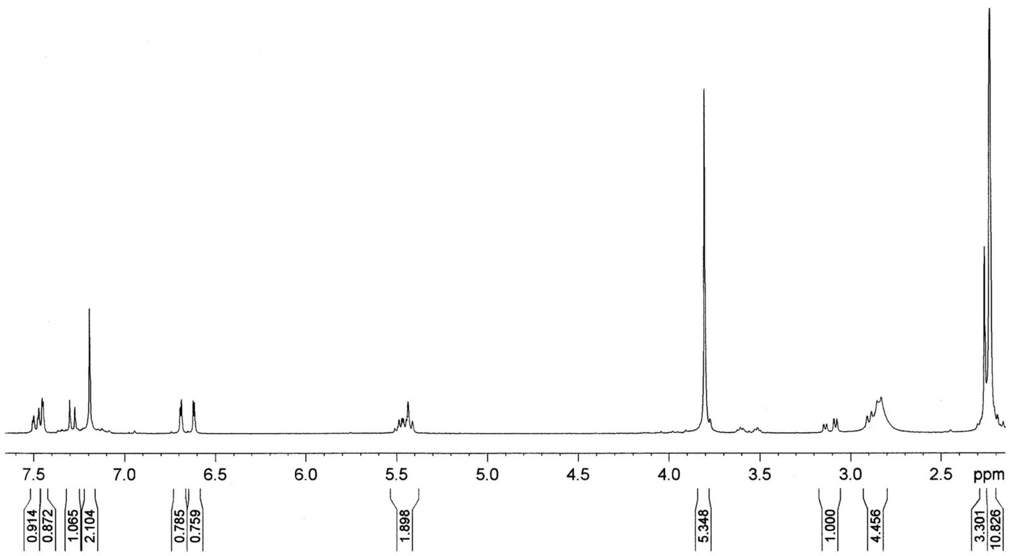

^{13}C-RMN spectrum of **12d**

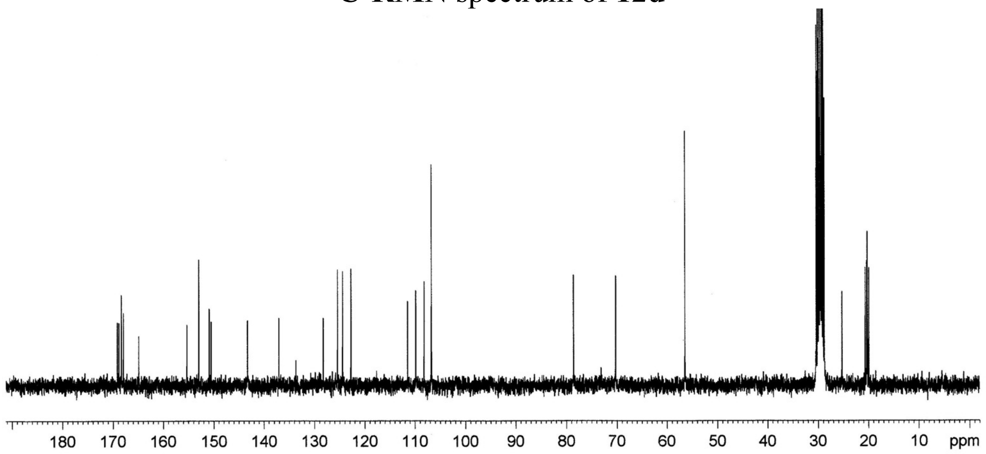

HMQC-RMN spectrum of **12d**

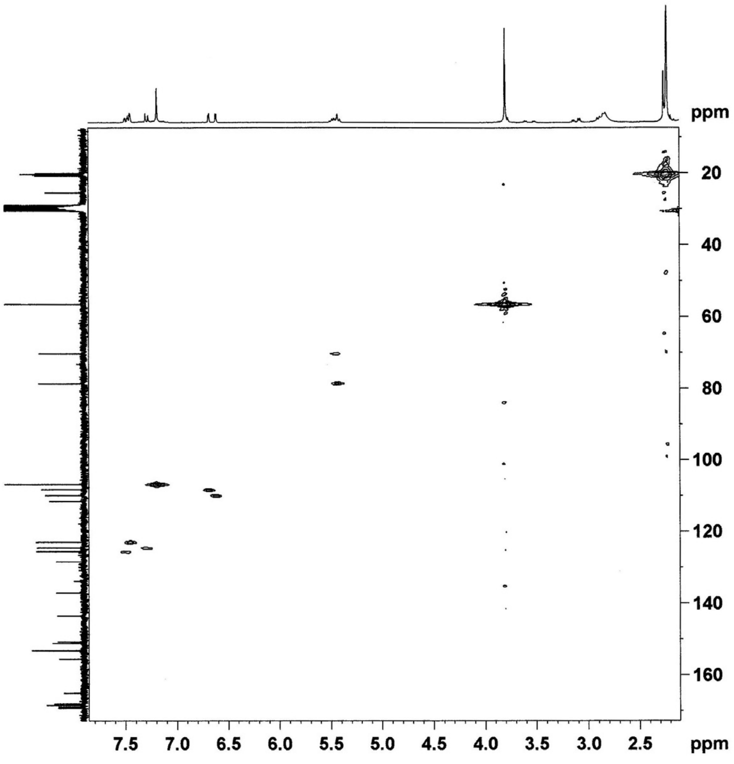

ESI-MS spectrum of **12d**

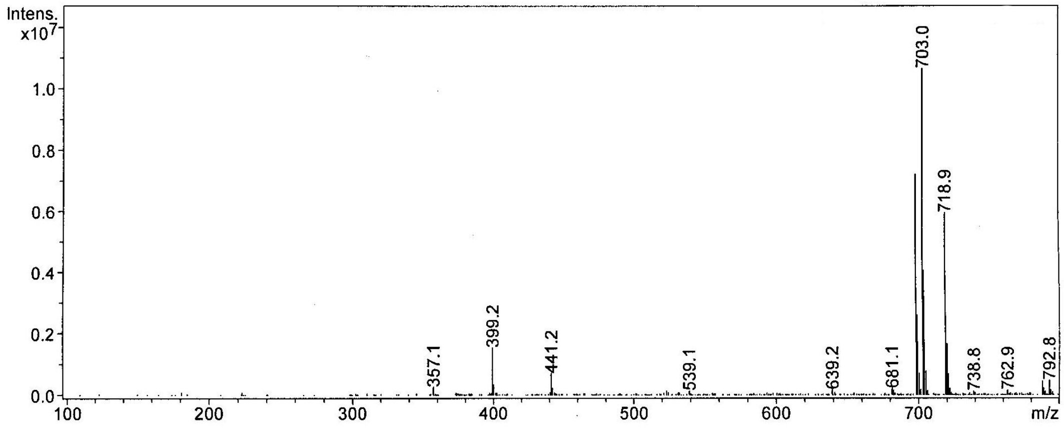

Figure S7. Spectra of 3-*O*-(4-hydroxy-3,5-dimethoxybenzoyl)-epicatechin (**13b**).

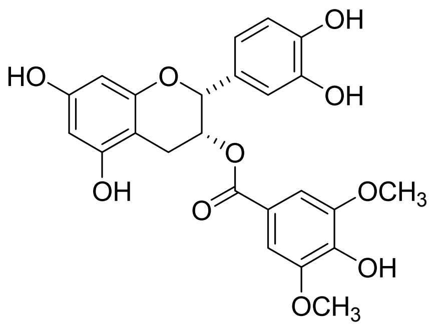

[1]**H-RMN** spectrum of **13b**

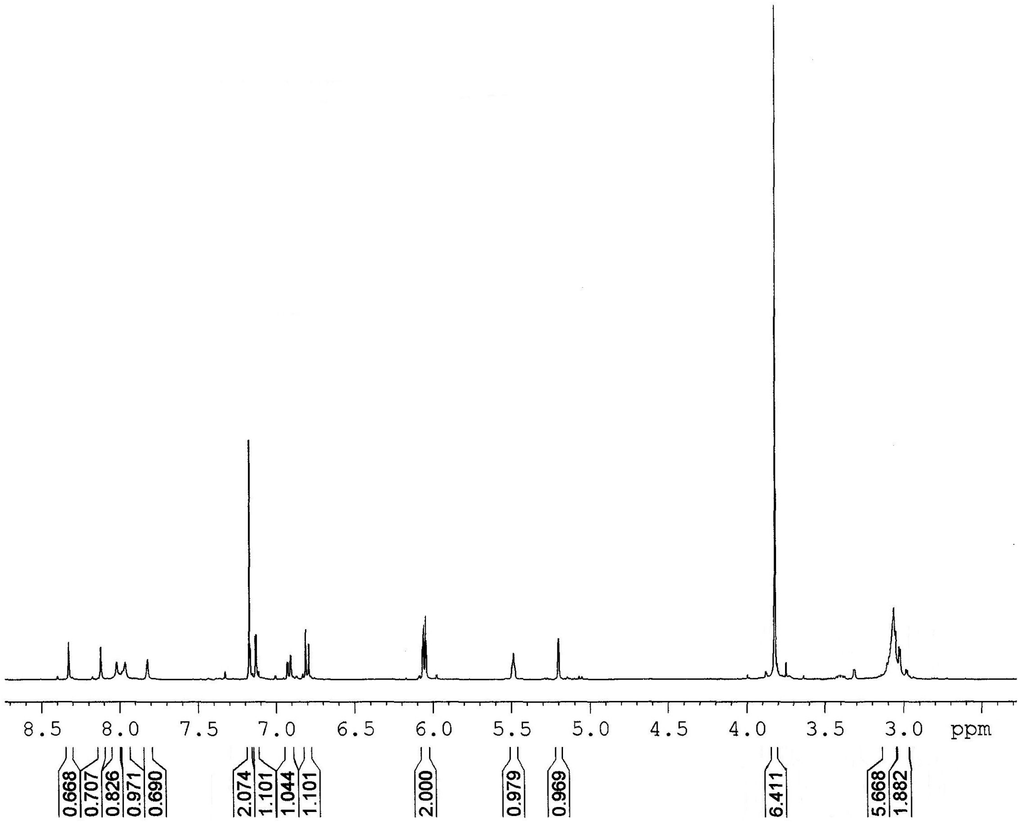

^{13}C-RMN spectrum of **13b**

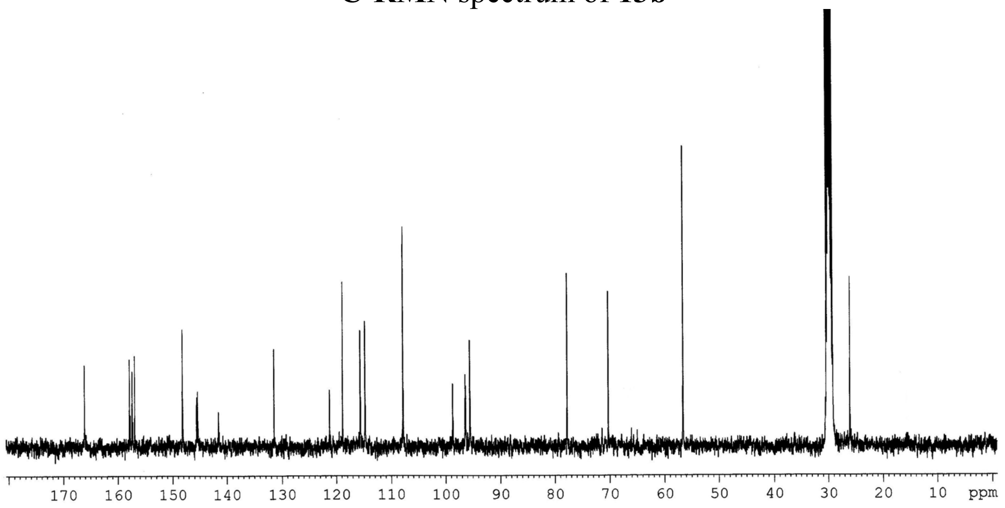

HMQC-RMN spectrum of **13b**

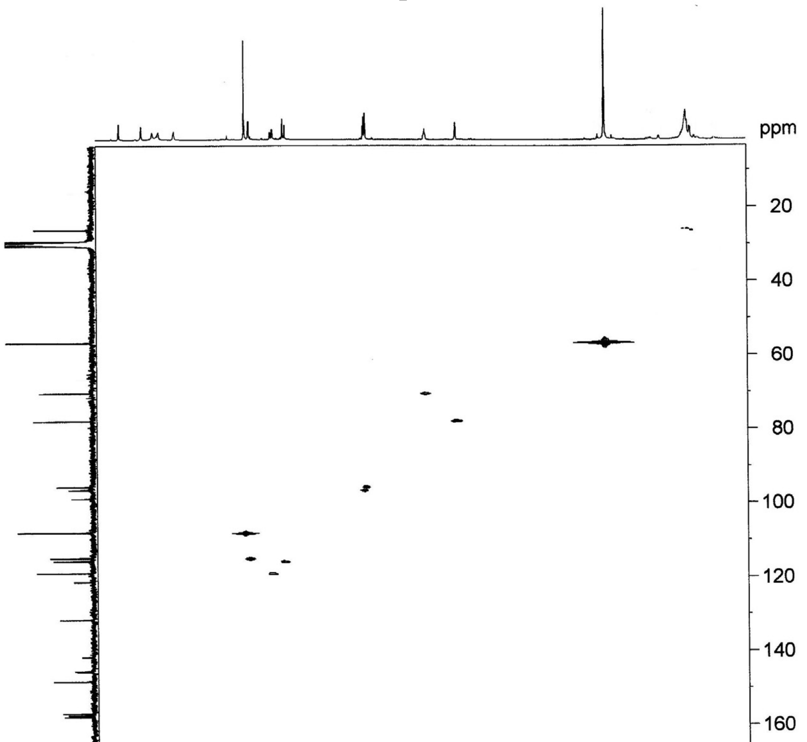

ESI-MS spectrum of **13b**

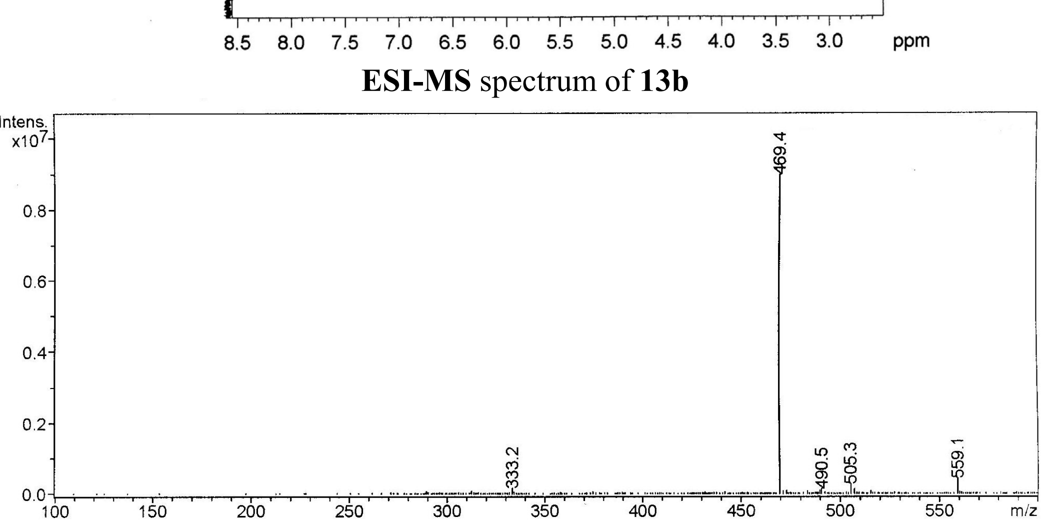

Supplement: Supplementary file 1 [file molecules-18-08319-s001.pdf]
